# Structure–Reactivity Relationships in a Small Library of Imine-Type Dynamic Covalent Materials: Determination of Rate and Equilibrium Constants Enables Model Prediction and Validation of a Unique Mechanical Softening in Dynamic Hydrogels

**DOI:** 10.1021/jacs.4c08099

**Published:** 2024-10-01

**Authors:** Francis L. C. Morgan, Ivo A. O. Beeren, Jurica Bauer, Lorenzo Moroni, Matthew B. Baker

**Affiliations:** † Department of Instructive Biomaterials Engineering, MERLN Institute for Technology-Inspired Regenerative Medicine, Maastricht University, 6229 ER Maastricht, The Netherlands; ‡ Department of Complex Tissue Regeneration, MERLN Institute for Technology-Inspired Regenerative Medicine, Maastricht University, 6229 ER Maastricht, The Netherlands

## Abstract

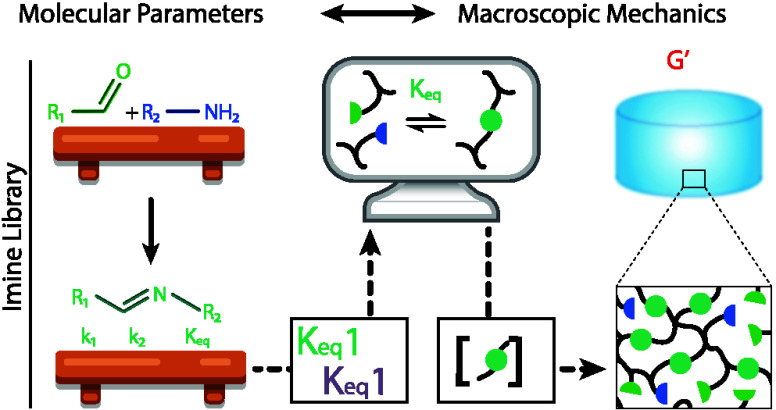

The development of next generation soft and recyclable materials prominently features dynamic (reversible) chemistries such as host–guest, supramolecular, and dynamic covalent. Dynamic systems enable injectability, reprocessability, and time-dependent mechanical properties. These properties arise from the inherent relationship between the rate and equilibrium constants (RECs) of molecular junctions (cross-links) and the resulting macroscopic behavior of dynamic networks. However, few examples explicitly measure RECs while exploring this connection between molecular and material properties, particularly for polymeric hydrogel systems. Here we use dynamic covalent imine formation to study how single-point compositional changes in NH_2_-terminated nucleophiles affect binding constants and resulting hydrogel mechanical properties. We explored both model small molecule studies and model polymeric macromers, and found >3-decade change in RECs. Leveraging established relationships in the literature, we then developed a simple model to describe the cross-linking equilibrium and predict changes in hydrogel mechanical properties. Interestingly, we observed that a narrow ≈2-decade range of *K*_eq_’s determine the bound fraction of imines. Our model allowed us to uncover a regime where adding cross-linker before saturation can decrease the cross-link density of a hydrogel. We then demonstrated the veracity of this predicted behavior experimentally. Notably this emergent behavior is not accounted for in covalent hydrogel theory. This study expands upon structure–reactivity relationships for imine formation, highlighting how quantitative determination of RECs facilitates predicting macroscopic behavior. Furthermore, while the present study focuses on dynamic covalent imine formation, the underlying principles of this work are applicable to the general bottom-up design of soft and recyclable dynamic materials.

## Introduction

Dynamically cross-linked material systems^1^ have emerged as promising platforms for self-healing polymers,^2,3^ chemical recycling platforms,^4,5^ and biomedical materials.^6,7^ Dynamic bonds impart time-dependent properties to materials through the explicit relationships between binding dynamics–described by rate, *k*_1_ and *k*_–1_, and equilibrium, *K*_eq_, constants (RECs)—and the resulting macroscopic mechanical properties. The ability to tune RECs through molecular engineering and consequently modulate the resulting mechanical properties has underpinned the broad success of dynamic materials.^8,9^ While the capacity to target specific mechanical regimes by tuning exchange dynamics has been established,^10,11^ there remain few reports of complete sets of RECs and their impact on materials properties for a given dynamic system. This lack of fundamental data has hindered the development of models for predicting bulk mechanical properties from REC data.^12,13^ Consequently, there is a strong need for quantified RECs of dynamic systems to enable a paradigm shift from dynamic binding as a useful trend, to dynamic binding as a powerful predictive tool in the design of novel materials.

The relationships between molecular parameters such as RECs and macroscopic mechanics is general to dynamic systems,^9,14−16^ and has been used to create materials from boronic acid esters,^9^ supramolecular host–guest cucurbiturils,^12,15,17^ and dynamic covalent imine formation.^18,19^ In addition to the utility in controlling formation kinetics (∝ *k*_1_), bulk stiffness (∝ *K*_eq_), flow (∝ *k*_–1_, *K*_eq_), and longevity (∝ *k*_1_, *K*_eq_),^8^ dynamic binding has important implications in cell-adhesive ligand interactions and biomolecule presentation for biomedical applications.^20,21^ The fields of tissue engineering^7,22,23^ and mechanobiology^21,24^ have also incorporated dynamic chemistry into soft (bio)materials to overcome the often-conflicting^25,26^ mechanical properties required for hydrogel processing and targeting a biomechanical niche.

Schiff base (imine) cross-linking is a commonly used dynamic covalent chemistry, with examples in biomedical materials,^19,27,28^ recyclable and biobased vitrimers,^29−31^ and bioelectronics.^32,33^ Among dynamic imines, the formation of hydrazones and oximes are commonly investigated reactions, likely in part since they can benefit from the modification strategies previously developed in the field of chemical biology.^34,35^ Despite decades of oxime (and hydrazone) ligation research using model small molecule systems, the full set of RECs are rarely reported in the same work. Kölmel and Kool prepared a comprehensive review covering hydrazone and oxime bioconjugation studies.^35^ While much useful information on relative reactivity and trends can be obtained from this seminal body of work, how reactivity measured in model studies translates to the reactivity of more complex polymeric systems and materials remains unclear. Additionally, while some existing covalent network models have been adapted to represent the reversible nature of dynamic boronic acid ester cross-links,^36,37^ and more recently Marco-Dufort et al. studied the relationship between RECs and macroscopic mechanics in telechelic boronic acid ester hydrogels,^9^ we lack distinct network models for dynamic networks composed of reversible junctions.

**Figure 1:**
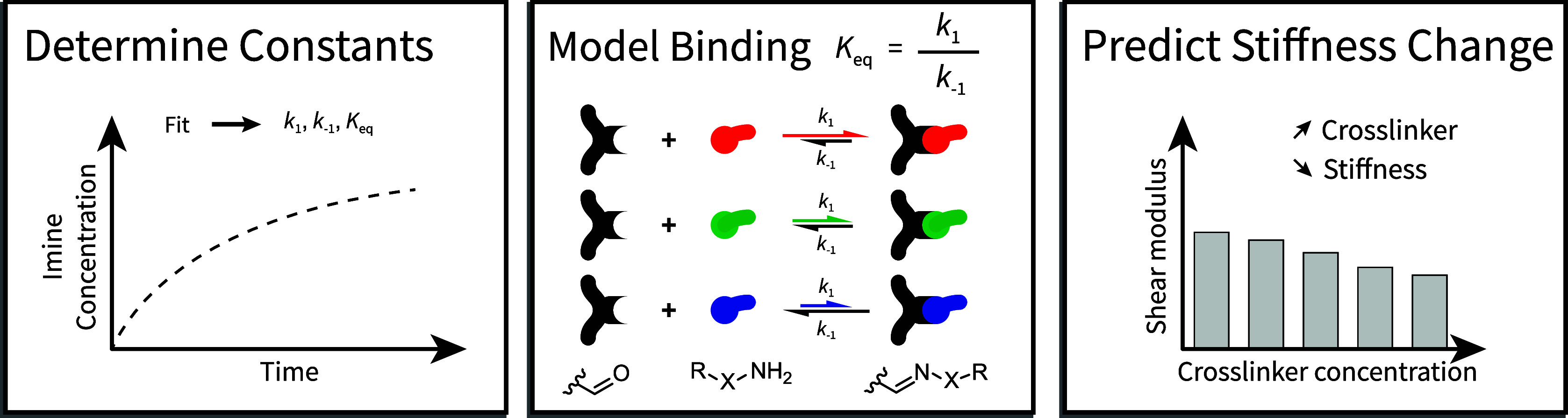
Linking rate and equilibrium constants (RECs) to macroscopic mechanics via experimental determination and application of a binding model. There is a fundamental relationship between RECs of dynamic covalent chemical reactions, and the resulting bulk macroscopic mechanical properties of soft matter that use dynamic covalent reactions to form their junctions. By combining experimentally determined RECs with models that describe changes in molecular junction (cross-link) concentration, we were able to predict, and then experimentally validate, a competitive-softening regime from mixed dynamic cross-linkers.

In this work, we aim to build upon the structure–reactivity relationship that imines display with their component amines and aldehydes (Figure 1). First, we selected a series of amines with single point atomic variations to explore how molecular variation affects the reactivity of amines for dynamic imine formation. Then, we assessed the theoretical magnitude of differences in electronic behavior and reactivity trends through simplified Density Functional Theory (DFT) calculations for this series. Next, we quantified the *k*_1_, *k*_–1_ and *K*_eq_ of our amines when reacting with different aldehydes to compare theoretical and experimental trends in reactivity. By using a model small molecule aldehyde, an oxidized polysaccharide, and a synthetic polymer with pendant aldehyde groups, we also highlighted the effect natural and synthetic polymeric systems can have on aldehyde reactivity. These differences in reactivity are critical to understanding how existing literature values for molecular parameters of small molecules translate to practical polymeric material systems.

The general relationships between RECs and macroscopic mechanics have been established in literature, and the RECs measured in the current work match our previous experimental work.^19^ To build upon our previous study, we were interested to see if we could tease out more complex relationships in these materials with concrete numbers in hand. For example, what happens if cross-linkers are in competition? Consequently, to illustrate how quantitative determination of molecular parameters facilitates the prediction of macroscopic mechanical properties, we employ a reversible binding model to predict and validate changes in hydrogel stiffness for competitively cross-linked oxidized alginate-based hydrogels. Notably, these predictions reveal a new responsive regime accessible only to dynamic systems using competitive reversible cross-linking. This result underscores the broader spectrum of molecular behaviors accessible to dynamic systems compared to traditional covalent systems. Additionally, our model highlights that the majority of changes in the bound fraction of cross-linking function occur in a small ≈2 decade window of *K*_eq_’s, challenging the prevalent idea that access to a broad range of *K*_eq_’s is necessary to access a broad range of mechanical properties. Furthermore, we argue that this emergent behavior highlights the need for new theories specific to dynamic systems, to capitalize on the relationship between their molecular and macroscopic properties. Overall, this work will be of interest to the broader community of materials chemists working with functional soft materials, for the rational, bottom-up design of specific macroscopic mechanical regimes for a spectrum of applications.

## Results and Discussion

### Selection of Amine and Aldehyde Molecules to Study the Molecular Parameters of Schiff Base Formation

**Figure 2:**
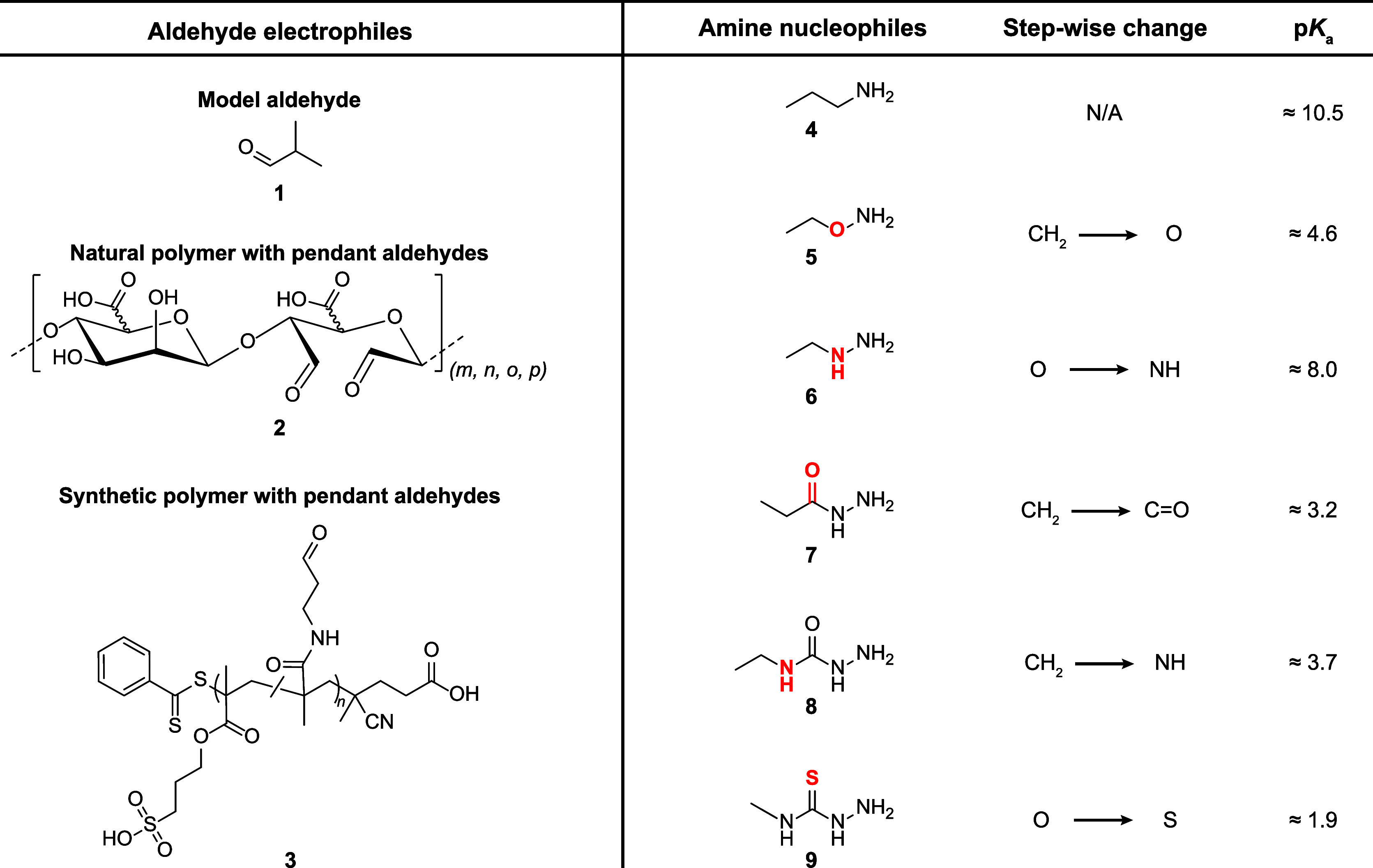
Chosen amine and aldehyde molecules to study the RECs of Schiff base formation. (Left) Three aliphatic aldehydes chosen for this study. Isobutyraldehyde (**1**), as a model compound, oxidized alginate (**2**), as representative of the oxidized natural polysaccharides commonly used as biomaterials, and a synthetic aldehyde polymer (**3**), as a synthetic and well-defined system for comparison.^38^ (Right) Series of amine nucleophiles (**4**–**9**) chosen for this study. Starting from propylamine, we can make a series of incremental single-point changes (highlighted in red) to the reactive group to highlight how even a single atomic change can have significant changes on the RECs (vide infra). The p*K*_a_ values are for the conjugate acid of each amine and are taken from literature (**4**;^39^**5**;^40^**6**;^41^**7**;^42^**8**;^43^**9**^44^). These p*K*_a_ values are given as approximate due to slight differences in the reported temperatures and salinities compared to our study.

To study the range of rate and equilibrium constants accessible through variation of the amine group, we selected a series of amines that can be described by single-point atomistic changes, starting from a primary aliphatic amine as a point of reference (Figure 2). This library was designed to discern the effect that even a single atom in the nucleophile’s structure can have on reactivity, and includes both common (hydrazide, hydroxylamine) and uncommon (hydrazine, thiosemicarbazide) amine partners for imine formation.

In selecting aldehyde partners, we wanted to study the effect of binding in both model (small molecule) and polymeric (as used in hydrogels) aldehydes. To this end, we selected a model aliphatic aldehyde, an oxidized polysaccharide and a synthetic polymer with pendant aldehyde groups (Figure 2). After testing several aliphatic aldehydes, only isobutyraldehyde and cyclohexanecarboxaldehyde proved stable under aqueous conditions without complex secondary structural forms (Figure S1). Of these two, we chose isobutyraldehyde, as cyclohexancarboxaldehyde is sterically constrained, and less representative of the aliphatic aldehyde groups found in most polymeric systems.^45−48^ Oxidized alginate was chosen as our natural polymer backbone as it is commonly used in biomedical applications, accessible, affordable, and has previously shown efficient hydrogel formation via imine cross-linking.^19,49^ While synthetic polymers containing pendant aldehydes are uncommon, we recently reported and used a well-defined copolymer with pendant aldehyde groups.^38^ This copolymer is referred to as pSM-*co*-OMAm and its structure (**3**) is given in Figure 2.

### Exploring a Simplified Density Functional Theory Approach to Model Trends in Amine Reactivity toward Imine Formation

Quantum chemical calculations can be a powerful tool for the determination of molecular properties. A key advantage of computational methods lies in their potential for screening scenarios to determine structure–property relationships, often at a lower cost than wet-lab experimental determination.^50−52^ Here, we set out to implement DFT calculations to assess the differences in molecular descriptors of our chosen amine nucleophiles, and determine if these simplified descriptors could allow us to capture observed experimental trends.

To determine the molecular descriptors we our series of amine nucleophiles, we performed DFT calculations at the B3LYP/6-31G+(d,p) level of theory. The determined molecular descriptors are compiled in Table 1. We found that all amines are predicted to react spontaneously with acetaldehyde (negative Δ_r_*G*°) from a thermodynamic perspective. Furthermore, comparing the Δ_r_*G*° of our amines, we would expect hydrazone (**7**) to have a much smaller equilibrium constant (as Δ_r_*G*° = -*RT* ln *K*_eq_) than semicarbazone (**8**) and oxime (**5**), which aligns with previous reports.^35^ Considering Δ*G*_2_* as proportional to *k*_1_ (See “Comparison of trends between calculated Gibbs free energies and experimentally determined rate and equilibrium constants” and Figure S2), we also find that **7** is expected to react more slowly than **8** and **5**, which is consistent with our previous experimental work.^53^ We also determined the natural charge on the terminal (nucleophilic) nitrogen atom and the nucleophilicity index for each amine.^54^ Here, we observe a general increase in nucleophilicity as the natural charge becomes more negative (Table 1, Figure S3), with a similar weak linear correlation between Δ*G*_1_*/*RT* and both the nucleophilicity and natural charge. This result indicates that a range of nucleophilicities is predicted across the series, giving confidence that the different electronic structures of the nucleophiles could provide differences in reactivity.

**Table 1:** Gibbs Free Energies for Imine Formation (Δ_r_*G*°), as Well as the Formation (Δ*G*_1_*) and Breakdown (Δ*G*_2_*) of the Tetrahedral Intermediate, for the Reactions between Amines **4–9** and Acetaldehyde (AA) as Calculated by DFT Using the B3LYP/6-31G+(d,p) Basis Set

amine	*N*^a^ (eV)	nat. charge^b^ (e^–^)	imine^c^	Δ*G*_1_*^d^ (kcal·mol^–1^)	Δ*G*_2_*^e^ (kcal·mol^–1^)	Δ_r_*G*°^f^ (kcal·mol^–1^)
4	2.887	–0.960	4+AA	10.73	–11.61	–0.87
5	2.589	–0.634	5 + AA	9.01	–16.78	–7.77
6	2.903	–0.774	6 + AA	10.89	–18.02	–7.13
7	2.683	–0.747	7 + AA	6.39	–6.94	–0.54
8	2.828	–0.761	8 + AA	8.53	–16.39	–7.86
9	3.116	–0.754	9 + AA	10.54	–16.24	–5.70

a The nucleophilicity parameter (***N***) is a means to describe the relative strength of nucleophiles and was calculated according to *N* = 20(*I* – *A*)/*I*^2^, where *I* is the vertical ionization energy approximated as *I* ≈ *E*_HOMO_ while *A* is the electron affinity approximated by (*A* ≈ *E*_LUMO_).^54^

b The calculated natural change is the inherent charge (in electrons, where one electron is equal to a charge of −1) present on the terminal (reacting) nitrogen atom as determined by DFT.

c The imines used for DFT calculations were performed using acetaldehyde (AA) as a model aldehyde.

d Gibbs free energy for the formation of the tetrahedral intermediate.

e Gibbs free energy for the breakdown of the tetrahedral intermediate to form an imine.

f Gibbs free energy for the overall reaction of imine formation from acetaldehyde and the given amine.

**Scheme 1:**
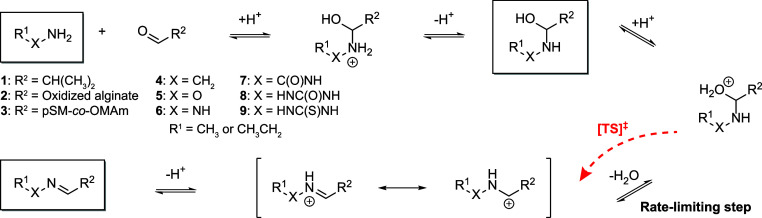
Standard Mechanism of Imine Formation from an Amine and Aldehyde At physiological pH, the accepted standard mechanism for imine formation is described according to this scheme. R^1^ and X describe the library of imines while R^2^ describes the selected aldehydes used in our experimental study. The rate-Limiting step (and accompanying transition state–highlighted in red) for imine formation is the dehydration of the tetrahedral intermediate. The boxed structures indicate the structures that were selected for DFT modelling.

In order to obtain more insight, we also attempted to analyze a simplistic reaction pathway using acetaldehyde as the aldehyde to limit the computation load. Only the more reactive free (nonhydrated) aldehyde was considered, as the exchange dynamics between the hydrated acetal form (under aqueous conditions) and free forms are rapid.^55^ Considering the putative reaction mechanism for imine formation under physiological conditions (Scheme 1), the rate-limiting step is the dehydration of the tetrahedral intermediate.^56^ To simplify the calculations, we chose to model the nonprotonated tetrahedral intermediate and compare this to the starting amine/aldehyde and final imine product in implicit solvent conditions. The obtained values were then used to approximate the Δ_r_*G*°, Δ*G*_1_*, and Δ*G*_2_*, corresponding to the Gibbs free energies of reaction, formation of the tetrahedral intermediate, and breakdown of the tetrahedral intermediate, respectively (Table 1, Figure S2). Given the approximations made in this approach, the values reported here should not be treated as absolute. Previous studies on hydrazone hydrolysis^57^ and the effect of salinity^58,59^ have established that the rate-limiting step is both ion and solvent assisted. To simplify the energy landscape, we assume that any change in transition state energies derived from explicit water molecules or ions is similar across the series, and consequently, has limited impact on general trends (Figure S2). A similar approach was used recently to probe the equilibrium for boronic acid ester formation.^9^ Knowing that we had molecules in hand with different expected trends in reactivity, we next wanted to see how this was reflected in experimentally determined rate constants.

### Experimental Methods for Determining the Rate and Equilibrium Constants of Dynamic Schiff Base Formation

**Figure 3:**
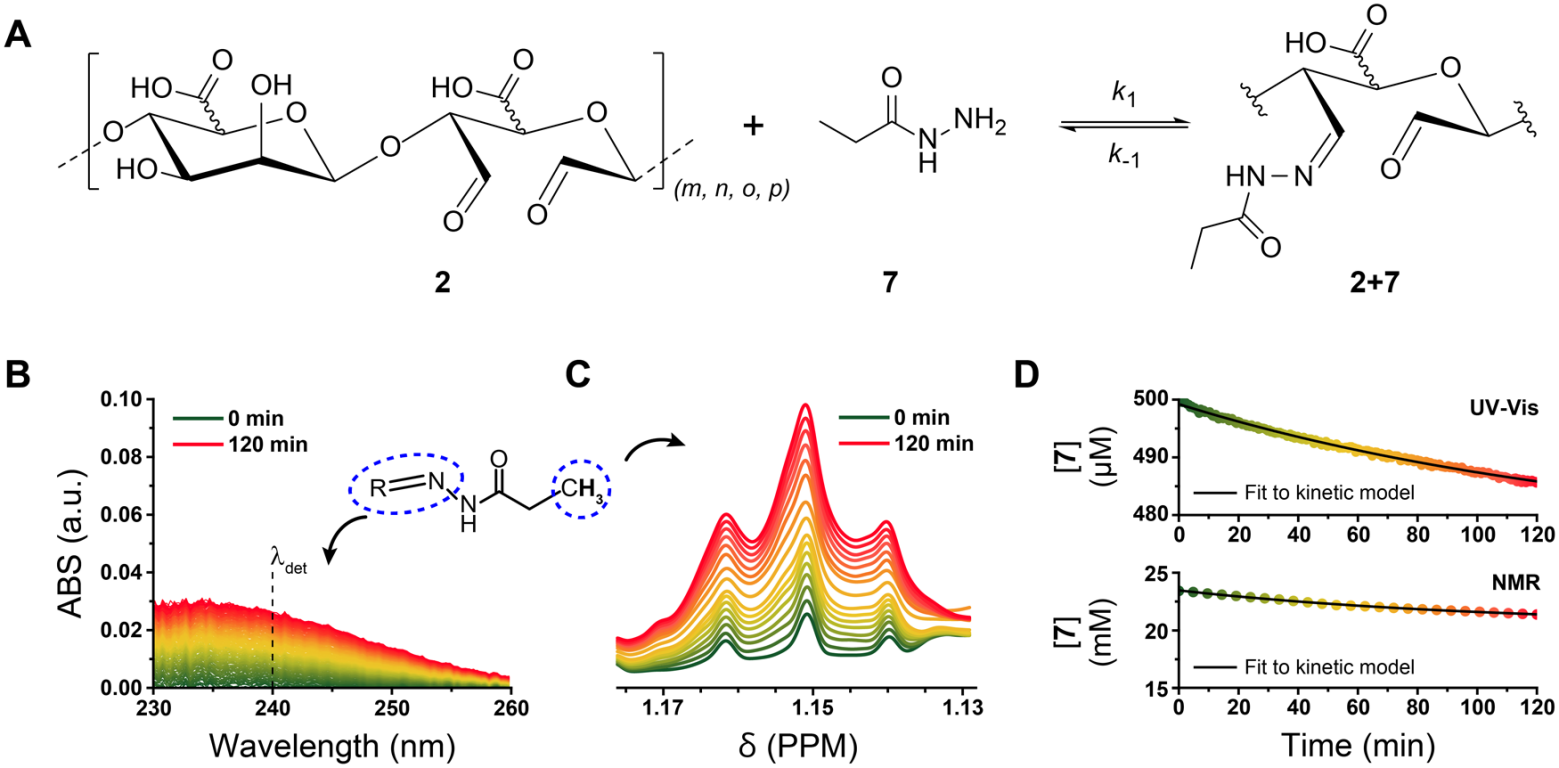
Comparing the determination of RECs via NMR and UV–vis spectroscopy. (A) Schematic example of imine formation between aldehyde **2** and amine **7** to form imine **2** + **7**. Example of a raw kinetic measurement using UV–vis (B) and NMR (C) spectroscopy. In both methods, the formation of the imine is measured and subsequently subtracted from the initial amine concentration to give the consumption of amine over time. (D) The resulting kinetic traces with accompanying fits to the consumption of **7** using eq 5 (See Materials and Methods), a reversible second order bimolecular equimolar model developed by Dirksen et al.^61^ The experimental conditions and obtained kinetic parameters are presented in Table 2.

Prior to determining the rate and equilibrium constants for our full series of amines, we compared both UV–vis and NMR spectroscopies as possible methods for kinetic data acquisition (Figure 3). We chose to compare these methods using oxidized alginate (**2**) with hydroxylamine (**5**) and hydrazide (**7**) as both the resulting oxime and hydrazone linkages are commonly used with oxidized biopolymers (Figure 3A). Example plots of imine double bond absorbance^60^ (UV–vis) and terminal-methyl peak intensity (NMR)are shown in Figure 3B,C. Both methods resulted in clear evolving signals that we could use to follow the production of imine over time, with no obvious difference between the relative growth rates of imine signal despite the different signal origin (double bond vs methyl).

Fitting kinetic data to a model for the determination of rate constants is commonly done using pseudo-first order approximations due to their ease of use and implementation. Similarly, determination of equilibrium constants is typically done via titration experiments. However, these approaches only give a single molecular parameter, rather than the full complement of RECs we desired for bottom-up design of dynamic soft materials. Here, we employed a reversible second order bimolecular equimolar model,^61^ which enables the simultaneous determination of both *k*_1_, and *k*_–1_—and consequently *K*_eq_, as *K*_eq_ = *k*_1_/*k*_–1_. This model fits the consumption of the amine over time; here, we determine the amine concentration by subtracting the measured imine concentration from the known initial amine concentration. The resulting conversions to plots of concentration over time and accompanying fit to a kinetic model can be seen in Figure 3D.

We first followed the formation of oxime and hydrazone by UV–vis. The absorbance signal for imines was quite low (<0.1 a.u.), with a maximum close to the solvent cutoff wavelength (Table 2). Consequently, we chose 240 nm as the detection wavelength for imine formation to avoid contributions from background solvent noise. We found the second order rate constant (*k*_1_) for oxime (**2** + **5**) formation to be 0.058 L·mol^–1^·s^–1^, which is lower than previously reported values for model studies of oxime formation.^62,63^ However, these studies use aromatic aldehydes to facilitate quantification, an excess of one reagent, and varying salt/buffer conditions. In comparison to oxime formation, the measured *k*_1_ for hydrazone formation (0.009 L·mol^–1^·s^–1^) was approximately 6-fold smaller. This slower relative rate is consistent with previous reports.^35,58^ The measured reverse rate constants (*k*_–1_) were similar for both hydrazone and oxime (52 × 10^–6^ s^–1^ vs 91 × 10^–6^ s^–1^), with resulting *K*_eq_^’^s 2–5 orders of magnitude lower than those typically seen in model systems for hydrazone (10^2^ L·mol^–1^ vs 10^4^–10^6^ L·mol^–1^) and oxime (10^3^ L·mol^–1^ vs 10^8^ L·mol^–1^), respectively.^34^ These lower *K*_eq_ values are discussed in the next section.

**Table 2:** Comparing the Rate and Equilibrium Constants Obtained for the Reaction between Amines **5** and **7** with Oxidized Alginate (2) via NMR and UV–Vis Spectroscopy^d^

method	imine	[salt]_total_ (mM)	[2]_0_ (mM)	[7]_0_ (mM)	*k*_1_ (10^–2^·L·mol^–1^·s^–1^)	*k*_–1_ (10^–6^·s^–1^)	*K*_eq_^a^ (10^3^·L·mol^–1^)
UV–vis	2 + 5	159^b^	0.5	0.5	5.8 ± 0.2	52 ± 25	1.3 ± 0.6
UV–vis	2 + 7	159^b^	0.5	0.5	0.9 ± 0.2	91 ± 12	0.10 ± 0.01
NMR	2 + 5	57^c^	5	5	0.5 ± 0.00(1)	29 ± 1	0.19 ± 0.00(6)
NMR	2 + 7	57^c^	24	24	0.09 ± 0.00(3)	119 ± 9	0.007 ± 0.00(1)

a The equilibrium constant (*K*_eq_) is calculated as *k*_1_/*k*_–1_.

b The total concentration of salts in the cell culture Phosphate Buffered Saline (PBS) used as a solvent. Values taken from supplier’s data sheet.

c Concentration of phosphate salts used in the preparation of buffered D_2_O. See Materials and Methods.

d Reported values for *k*_1_ and *k*_–1_ are the mean ± standard deviation of 2 replicates with the exception of NMR values for **2** + **7** which are the value ± the standard fit error for a single replicate. Molar absorptivity coefficients used for conversion from absorbance to concentration are reported in Table S1.

Next we studied oxime and hydrazone formation by NMR and immediately noticed that the *k*_1_^’^s were consistently 1 order of magnitude smaller than the corresponding *k*_1_^’^s obtained by UV–vis, yet maintained the same relative difference in rates of oxime vs hydrazone formation (Table 2). Notably, these NMR experiments were performed at a reduced salt concertation, since higher salt concentrations often lead to reduced signal.^64^ This change in measured rate constant is likely an effect of this different in salt concentration between the two methods, as it has previously been found that a higher salt concentration leads to a faster *k*_1_ and consequently, a higher *K*_eq_.^58,59^ We compared the effect of salt on **2** + **5** in Figure S4 using UV–vis and found a phosphate buffered D_2_O reduced *k*_1_ by a factor of 2 in the UV–vis measurements. The reverse rate constants (*k*_–1_) measured by NMR were relatively unaffected by the difference in salinity, with values similar to those found via UV–vis (29 × 10^–6^ s^–1^ and 119 × 10^–6^ s^–1^). The resulting *K*_eq_^’^s obtained via NMR decreased by approximately 1 order of magnitude in accordance with the 1 decade drop in *k*_1_.

Considering data acquisition via UV–vis and NMR, the rate and equilibrium constants obtained for oxime vs hydrazone exhibited the same relative differences, while the difference in the magnitude of obtained values between techniques could be attributed to the different measurement conditions. However, NMR measurements require much higher reactant concentrations (Table 2), and signal intensity is sensitive to salt concentration. NMR also generates fewer data points, which are time-averaged over the chosen number of scans—limiting time-resolution—but does have the advantage of directly measuring the imine species. In contrast, UV–vis allows reliable measurements under physiological salt concentrations, and has a lower detection limit. The main drawback of UV–vis is the underlying assumption that the evolving absorbance being measured arises exclusively from the imine—i.e. there must be no side reactions. We ultimately chose to proceed with UV–vis for subsequent kinetic data acquisition due to its salt tolerance and higher data point density for fitting.

### Effect of Aldehyde (1–3) and Amine (4–9) Structures on Rate and Equilibrium Constants

To enable the determination of imine concentrations via UV–vis for each amine–aldehyde pair, we first needed to determine the molar absorptivity coefficients (ε) and appropriate detection wavelengths (λ_det_) for each resulting imine. This was achieved using a combination of both NMR (imine concentration) and UV–vis (corresponding absorbance) spectroscopies at different concentrations, following by linear fitting to the Beer–Lambert Law (*A* = ε·*c*·*l*) where the slope corresponds to ε (light path length *l* = 1 cm). These results can be found in Figure S5 and Table S1. Then we set out to measure rate and equilibrium constants at equimolar concentrations for our selected series of amines and representative aldehydes via UV–vis.

Since we already had kinetic data for **2** + **5** and **2** + **7**, we first screened the remaining aldehydes for differences in reactivity using **5** and **7**. We found that the copolymer with pendant aldehydes (**3**) was the most reactive and subsequently used it to screen the accessible range of rate and equilibrium constants as a function of amine molecular structure (Table 3). The equimolar concentration of amine and aldehyde was adjusted where necessary to maintain an imine absorbance value in the linear regime (<1.0 a.u.) with sufficient signal; the low reactivity of **2** necessitated a higher concentration of 500 μM for a measurable signal, while the strongly absorbing **9** required decreasing the concentration to 50 mM.

**Table 3:** Rate and Equilibrium Constants of Dynamic Schiff Base Formation between Amine Aldehyde Backbones 1–3 and Nucleophiles 4–7 via UV–Vis Spectroscopy in PBS (pH = 7.4) at 20 °C for 2 h^j^

aldehyde	amine	conc.^a^ (μM)	ε^b^ (L·mol^–1^·cm^–1^)	*k*_1_ (10^–2^·L·mol^–1^·s^–1^)	*k*_–1_ (10^–6^·s^–1^)	*K*_eq_^c^ (10^3^·L·mol^–1^)
3	4	100	d	d	d	d
3	5	100	2200	950 ± 30	8.3 ± 5.1	1600 ± 1300
3	6	100	4200	1030 ± 30	365 ± 6	28 ± 1
3	7	100	8600	69 ± 3	57 ± 3	12.0 ± 0.7
3	8	100	9700	400 ± 20^e^^,^^f^	1.1 ± 0.3^e^^,^^f^	3700 ± 1100^e^^,^^f^
3	9	50^g^	14,200	75 ± 4^e^	11 ± 8^e^	87 ± 60^e^
2	5	500^h^	950	5.8 ± 0.2	52 ± 25	1.3 ± 0.6
2	6	100	2100	9.9 ± 2.2	450 ± 70	0.22 ± 0.06
2	7	500^h^	1870	0.9 ± 0.2	91 ± 12	0.10 ± 0.01
2	8	100	8700	12 ± 1	315 ± 11	0.39 ± 0.02
2	9	50^g^	7200	5.9 ± 0.0(3)^i^	i	i
1	5	100	2850	95 ± 8	106 ± 6	9.0 ± 0.5
1	6	100	4750	63 ± 2	160 ± 1	3.9 ± 0.1
1	7	100	11,100	12.4 ± 0.3	57 ± 1	2.2 ± 0.1
1	8	100	10,200	57.0 ± 1.5	76 ± 2	7.5 ± 0.4
1	9	50^g^	20,500	5.1 ± 0.3	5.2 ± 3.4	13 ± 7

a Equimolar concentration of both reactants at which the experiments were performed.

b Molar absorptivity coefficient for each imine measured via a combination of NMR and UV–vis spectroscopy (Table S1).

c The equilibrium constant (*K*_eq_) is calculated as *k*_1_/*k*_–1_.

d Amine **4** did not react with aldehyde **3** to form any product (Figure S6).

e The tabulated values were obtained by setting the *k*_1_ value obtained via irreversible 2nd order fitting, as a constant in eq 5 (See Materials and Methods) to constrain the fit by reducing the number of variables—see main text for Discussion.

f Values reported here are the global fit ± standard error instead of individual fits ± standard deviation for improved fitting.

g The concentration used was lowered to 50 mM to avoid signal saturation as **9** absorbs strongly.

h The concentration used was increased to 500 mM to increase signal intensity, compensating for the low reactivity of **2**.

i Constraining the fit for **2** + **9** as for **3** + **8** and **3** + **9** in “e” did not yield rational results; we suspect this reaction may be irreversible—and thus incompatible with eq 5—under the time scales and conditions we are probing (Figure S9, Table S2). The *k*_1_ value reported was obtained via irreversible 2nd order fitting.

j Reported values for *k*_1_ and *k*_–1_ are the mean ± standard deviation of 2–4 replicates.

Across the series, we observed *k*_1_^’^s spanning 0.7–10 L·mol^–1^·s^–1^ (≈1 decade) and *k*_2_^’^s spanning 1–360 s^–1^ (≈2 decades), with corresponding *K*_eq_^’^s spanning 12–3700 L·mol^–1^ (≈2 decades). Noticeably, the primary amine **4** did not yield any change in absorbance when paired with **3**. To confirm this observation, we acquired the ^1^H NMR of **3** + **4** after 16 h and could not observe imine peaks (Figure S6). This lack of reactivity can be explained by the high p*K*_a_ value of the conjugate acid of **4** (**≈**10.5)^39^ compared to the conjugate acids of the rest of our amine series (**5** ≈4.6;^40^**6** ≈8.0;^41^**7** ≈3.2;^42^**8** ≈3.7;^43^**9** ≈1.9).^44^ These p*K*_a_ values are given as approximate due to slight differences in the reported temperatures and salinities compared to our study. A p*K*_a_ over 3 units above the pH of our reaction leads to almost an exclusive population of the protonated (and thus unreactive) form of **4**, while all other p*K*_a_’s allow for a large population of nonprotonated (and thus reactive) amines.

Next, we took the series of reactive amines (**5**–**9**), and studied the effect that different aliphatic aldehyde structures (**1**–**3**) had on imine formation (Table 3). Considering their relative reactivity, it is clear that aldehyde **3** gives the highest *k*_1_^’^s and corresponding *K*_eq_^’^s, followed by **1**, and finally **2** (**3** > **1** > **2**). The lower reactivity of our model aldehyde (**1**) compared to **3** might be attributed to a proposed stabilization of the transition state by an 8-membered ring via hydrogen bonding from the amide group (Figure S7).^65,66^ Another contributing factor may be the steric effects from the tertiary carbon in **1**, which has been shown to induce a 3-fold decrease in apparent rate when reacting with phenylhydrazine.^66^ Meanwhile, aldehydes in oxidized alginate (**2**) are both sterically restricted by the polysaccharide backbone and largely in a hemiacetal state. Previous work has shown that these aldehydes exist in aqueous PBS in their hemiacetal form—stabilized in 6-membered rings by neighboring alcohol groups on the uronic acid residues.^48^ The hemiacetal form is further confirmed by the absence of free aldehyde peaks in the ^1^H NMR spectrum of **2** (Figure S8), and accounts for their lower relative reactivity.

**Figure 4:**
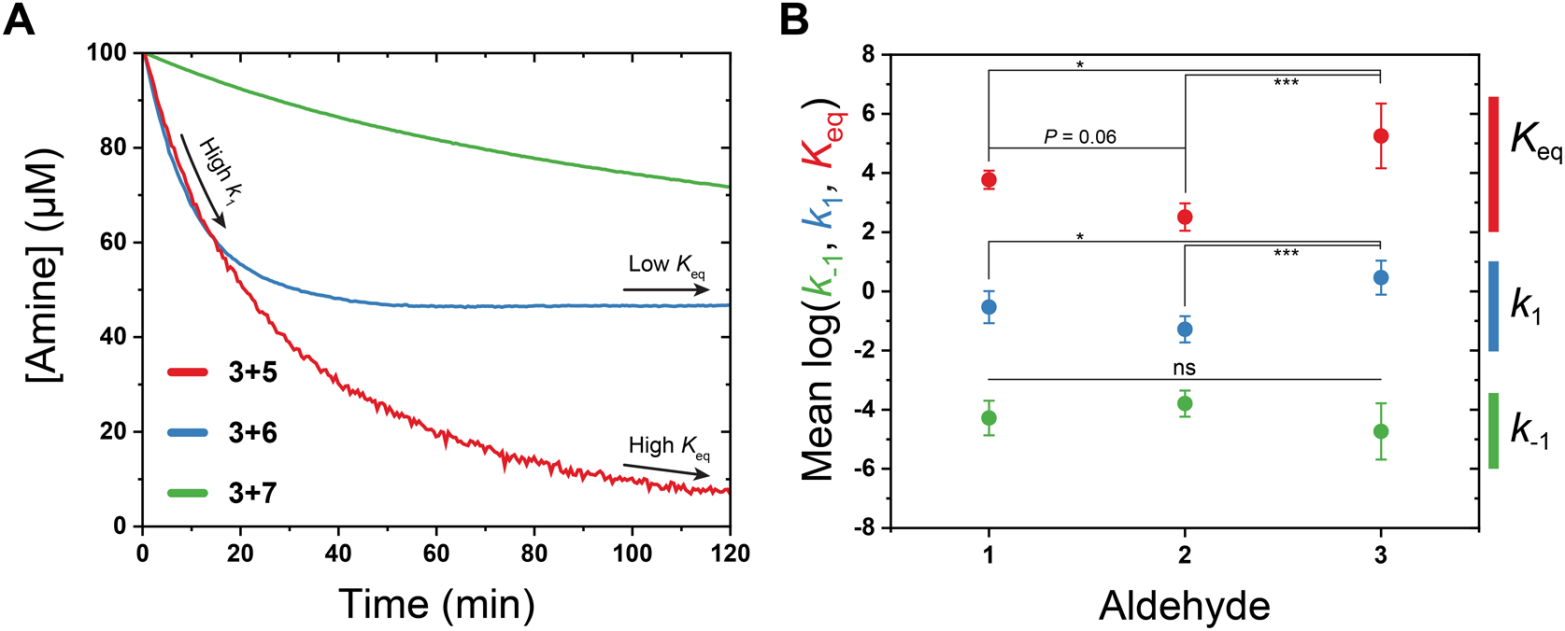
Visual illustration of the consumption of amines **5–7** when reacting with **3**, and comparison of average determined RECs grouped by aldehyde. (A) This visual comparison of amine consumption (prior to fitting) illustrates how hydrazine (**6**) has a similar high forward rate to hydroxylamine (**5**) yet rapidly attains a lower equilibrium constant similar to hydrazide (**7**). (B) Comparison of the mean RECs (circles, bars represent the standard deviation) for all aldehyde-amine pairs grouped by aldehyde to enable visual inspection of their relative magnitudes and spread. Statistical analysis was performed using a one-way ANOVA with Tukey’s multiple comparisons test: *P < 0.05, ***P < 0.0005.

Comparing the trends in reactivity for amines across the aldehyde series, we began by examining the kinetic traces prior to fitting. Interestingly, visual examination of the kinetic traces (presented as amine consumption for fitting, Figure 4A) highlights their different reaction profiles effectively. Considering **3** + **5**, **3** + **6**, and **3** + **7** as an example, this type of plot presents both a practical method for verifying the consistency of our fitted values (e.g., similar *k*_1_’s should have a similar initial rate of consumption prior to attaining a steady state regime; if *k*_1_ ≫ *K*_eq_ then reaction equilibrium is reached rapidly) and a clear illustration of the relative magnitudes of RECs. Overall, we saw that *K*_eq_’s followed the general trend of **7** < **6** < **8** < **5**, which is consistent with our previous observations on hydrogel stiffness and hydrogelation kinetics.^19,53^ In contrast, the *k*_1_’s showed a less consistent ranking of **7** < (**6** + **8** + **5**), with **7** as the slowest reacting, while **5**, **6** and **8** retained similar magnitudes but vary in order. One outlier to trends across amine reactivity was the behavior of **3** + **5** and **3** + **8**; while we can conclude that their rate was higher than that of **7**; the large error in these two fitted values precludes definitively ordering them within the series.

We also noticed while initially fitting **3** + **8**, **3** + **9** and **2** + **9** to eq 5 that the obtained *k*_–1_ (but not *k*_1_) values tended toward extremely small values resulting in large relative error of fit. To overcome this, we began by fitting these pairs to standard second order irreversible binding model and found well-fitting, matching *k*_1_’s (See Figure S9 and Table S2). Subsequently, we set these *k*_1_^’^s as constants in eq 5 to reduce the number of variables and improve the fit of *k*_–1_. This approach worked for **3** + **8** and **3** + **9**, but not for **2** + **9**. This finding suggests that **2** + **9** may in fact be irreversible under the explored conditions (and thus incompatible with our chosen model), though a much longer time scale would need to be probed to confirm this.

The ensemble of data, with variation in both aldehyde and amines, now spans 3 decades in *k*_1_, 2 decades in *k*_–1_, and 4 decades in the resulting *K*_eq_^’^s. While changing the aldehyde or the amine partner could lead to significant differences in the REC’s, we were also interested if globally one of the reaction partners had a larger influence on REC’s than the other. By plotting the mean of log-transformed RECs as a function of aldehyde partner, we started to uncover more general trends. We observed no statistically significant variation in *k*_–1_’s whereas the *k*_1_’s (and resulting *K*_eq_’s, as we calculated *K*_eq_ = *k*_1_/*k*_–1_) showed significant differences across aldehyde partners (Figure 4B). In contrast, a similar comparison of RECs as a function of amine shows no obvious trends (Figure S10). These observations suggest that globally, the chemical nature of the aldehyde has more influence than the amine over the rate and equilibrium constants for imine formation, and also that *k*_1_ is more sensitive to changes in the amine/aldehyde pair than the reverse rate constant.

A large diversity of RECs is important for targeting specific mechanical regimes through selected combinations of *k*_1_, *k*_–1_ and *K*_eq_ due to their relationships with mechanical properties (e.g., *k*_1_ ∝ gelation kinetics; *k*_–1_ ∝ stress relaxation/flow; *K*_eq_ ∝ bulk stiffness).^8^ For example, consider the three pairs **3** + **5**, **3** + **6** and **3** + **7**. In the context of bioprinting, a desirable material can rapidly cross-link (large *k*_1_), yet flow under stress (large *k*_–1_). Amines **5** and **7** are commonly used as dynamic cross-linker, indeed we previously studied mixing both **5** and **7** to obtain a balance between cross-linking kinetics (large *k*_1_) and rate of stress relaxation (large *k*_–1_).^19^Figure 4A is a clear display of how **3** + **6** possesses both a larger *k*_1_ akin to **3** + **5** and a larger *k*_–1_ similar to **3** + **7**. This interplay between the roles of different RECs also serves to illustrate why attaining explicit values for dynamic polymeric systems is paramount for the bottom-up design of dynamic systems for specific applications.

Another interesting observation in Figure 4B is the spread of molecular parameters for each aldehyde. The spread of *K*_eq_^’^s for the amine library with our model aldehyde (**1**) was narrow (≈0.5 decades), but increased to ≈1 decade (**2**) and ≈2.5 decades (**3**) when we move to polymeric systems, highlighting the different reactivity profiles of model vs polymeric aldehydes. It appears that the lower reactivity of oxidized alginate (**2**) is associated with compressed RECs into a smaller overall range, while the more reactive **3** had a larger spread, and thus accessible range, across *k*_1_, *k*_–1_ and *K*_eq_. Consequently, for the bottom-up design of dynamic covalent materials, we might expect more reactive aldehyde partners to offer a wider range of accessible mechanical regimes, though work on other systems needs to confirm the trend seen in our small library. We do note that further modification to the amines and aldehydes immediately adjacent to their reactive center can induce further differences in rate and equilibrium constants of several orders of magnitude.^63,67,68^ The ability to tune the reactivity of the amine and aldehyde independently offers further flexibility in attaining the desired combination of rate and equilibrium constants for a target application.

### Comparison of Trends between Calculated Gibbs Free Energies and Experimentally Determined Rate and Equilibrium Constants

**Figure 5:**
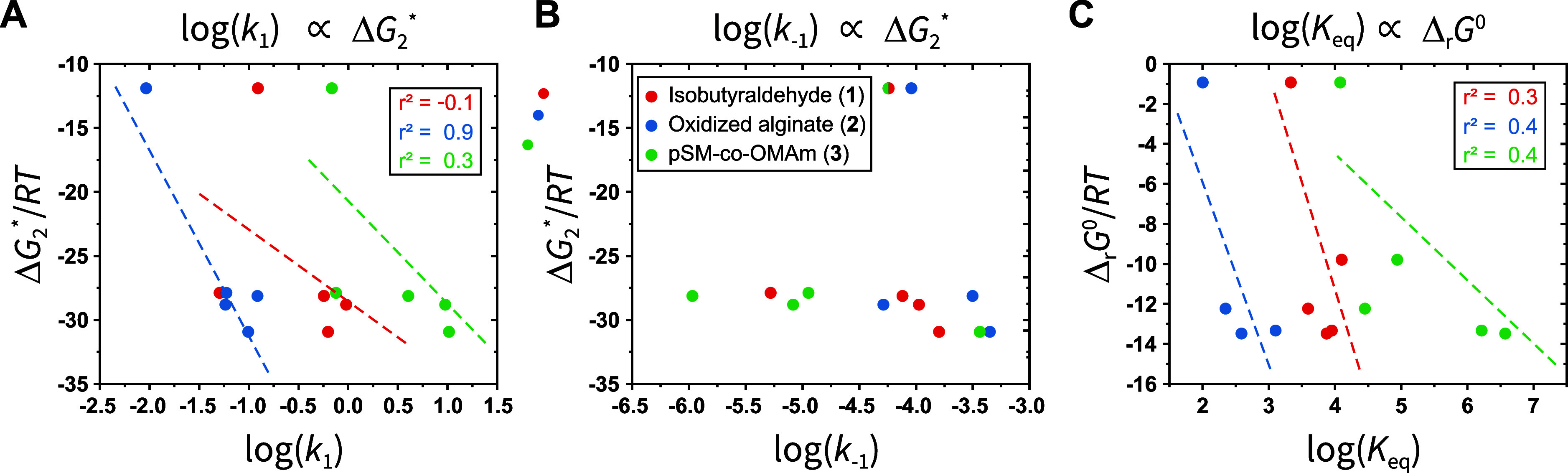
Semilog correlations between calculated DFT energy differences and experimental RECs. (A) Plotting Δ*G*_2_*/*RT* against log(*k*_1_) shows a weak linear trend in the expected direction; an increase in log(*k*_1_) as Δ*G*_2_*/*RT* becomes increasingly negative (larger magnitude). (B) No evident trend is seen between Δ*G*_2_*/*RT* and log(*k*_–1_), which is consistent with our simplified approach (Figure S2). (C) Plotting Δ_r_*G*^0^/*RT* against log(*K*_eq_) shows slightly stronger correlations between the Gibbs free energy of reaction and the measured equilibrium constants; as the absolute value of Δ_r_*G*^0^ becomes larger, we expect an increase in the associated equilibrium constant. The calculated Gibbs free energies are rendered dimensionless by division with *RT* to facilitate comparison and with the relevant logarithms of experimental rate values.

With both calculated molecular parameters and energy differences along with experimental rate and equilibrium constants, we wanted to investigate if there were any strong correlations between the different data sets. To do so, the calculated Gibbs free energies are rendered dimensionless by division with *RT* and then compared with the logarithms of experimentally determined RECs. We remind the reader that our determined Gibbs free energies assume that the known impact of explicit ion and solvent molecules^57−59^ are consistent across the series and do not affect general trends. Considering our simplified energy landscape (Figure S2), and by applying the Hammond postulate,^69^ we made the assumption that a more negative Δ*G*_2_* should lead to an increase in *k*_1_, via a corresponding decrease in activation energy of the rate-determining step by virtue of the transition state possessing more tetrahedral intermediate character (Figure 5A). According to this approach, a decrease in activation energy is concomitant with a more negative Δ*G*_2_*, which implies a similar correlation between a more negative Δ*G*_2_* and a decrease in *k*_–1_. However, this change in activation energy remains small relative to the magnitude of Δ*G*_2_* and consequently, we expect minimal correlation between Δ*G*_2_* and *k*_–1_ (Figure 5B). As usual, the *K*_eq_ is expected to correlate with Δ_r_*G*^0^ (Figure 5C). Ultimately, we observed only weak correlations (*r*^2^ < 0.5) that follow these expected trends, with the exception of **2** when comparing Δ*G*_2_*/*RT* to log(*k*_1_) (*r*^2^ = 0.9). We also verified that no clear trends were observed between the nucleophilicity or natural charge of our amine series and the corresponding *k*_1_’s (Figure S11), as these parameters are not involved in the rate-determining step of imine formation. Despite observing the expected trends, the precision and strength of these correlations is insufficient to enable robust in silico prediction of rate and equilibrium constants with our simplified approach. It is probably that our underlying assumption of a constant solvent and ion effect across the diverse amine structures explored in this work is an oversimplification. A potentially more accurate method for forecasting differences in reactivity would be to examine energies around the ion/solvent-assisted transition state of the rate-limiting step. However, these results are encouraging, in that a refined computational approach and larger data set could potentially yield stronger predictive power in the future, particularly with the advent of advanced artificial learning models,^50,51,70^ and enable the identification of dynamic covalent imine reactants for highly specific applications.

### Modeling Changes in Hydrogel Cross-Linked Fraction for Systems Composed of Competitive Imine Cross-Linkers

Previous work has shown that there can be correlations between RECs in dynamically cross-linked hydrogels and the bulk mechanical properties—creating the guidelines for reactivity–property relationships. To provide additional context, we have summarized works where differences in rate and equilibrium constants have successfully been exploited qualitatively to enhance material design (Table 4) and correlated to hydrogel mechanical properties. We also note that using a catalyst—like some of the examples in Table 4—affects rate constants without impacting equilibrium constants, which is orthogonal to varying rate and equilibrium constants through their chemical structure.^18^ There is a growing body of evidence in the literature that the modulation of RECs is a powerful tool for the rational design of hydrogels. Of note, the trends we have previously observed in hydrogel cross-linking and final modulus match well with our measured rate and equilibrium constants (Table 3), where *k*_1_’s (cross-linking rate) and *K*_eq_’s (final *G*′ after cross-linking) follow **5** > **8** > **7**.^53^

**Table 4:** Summary of Previous Work Demonstrating the Relationship between Rate and/or Equilibrium Constants for Imine Formation and Bulk Mechanical Properties in Hydrogels

aldehyde(s)		backbone	amine(s)	constant	property	ref
natural	aliphatic	oxidized alginate (2)	hydroxylamine (5), hydrazide (7), semicarbazide (8)	*K*_eq_, *k*_1_	*G*′, *t*_gel_	(53)
natural	aliphatic	oxidized alginate (2)	hydroxylamine (5), hydrazide (7)	*K*_eq_, *k*_–1_	*G*′,*G*(*t*)	(19)
synthetic	aliphatic	8-arm PEG	hydrazineylacetamide + ΔpH	*k*_1_,*k*_–1_	*t*_gel_,*G*(*t*)	(71)
synthetic	aliphatic, aromatic	8-arm PEG	hydrazineylacetamide	*K*_eq_, *k*_–1_	*G*′, *G*(*t*)	(46)
natural	aliphatic	hyaluronic acid	hydrazineylacetamide + catalyst	*k*_1_, *k*_–1_	*t*_gel_,*G*(*t*)	(72)
natural	aliphatic, aromatic	hyaluronic acid	hydrazineylacetamide	*K*_eq_, *k*_–1_	*G*′,*G*(*t*)	(73)
synthetic, natural	aliphatic	hyaluronic acid, 4-arm/8-arm PEG	hydrazineylacetamide + catalyst	*K*_eq_, *k*_–1_	*G*′,*G*(*t*)	(18)

To build upon the developing body of qualitative reactivity-property data in dynamic hydrogels, we wanted to use the RECs determined in this work to construct a simple model to predict the cross-linked fraction of aldehydes in a dynamic hydrogel as a function of the *K*_eq_ of imine formation. Such a model could enable the targeting of a specific degree of cross-linking (and thus mechanical properties) using dynamic imine reactions with known equilibrium constants and concentrations. By demonstrating the usefulness of a model to pair with explicitly determined molecular constants, we hope encourage future quantitative determination of RECs in dynamic systems, and take an initial step toward the development of robust predictive tools for dynamic systems.

In our recent work, we showed that mixed cross-linker systems are a useful approach to tune hydrogel mechanics.^19^ However, we remained curious as to what would happen when the competition of these cross-linkers for binding sites was perturbed. We decided to explore this scenario with a simplified model description. To do so, we began by considering the ideal maximum fraction of cross-linked aldehydes (χ_XL_^Ald^, where Ald refers to Aldehydes and XL denotes cross-linked) as a function of the molar ratio of added amine (relative to the aldehyde, χ_Am_) for a series of different *K*_eq_^’^s, which can be derived to be represented by eq 1 (See Supporting Information).
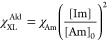
1

**Figure 6:**
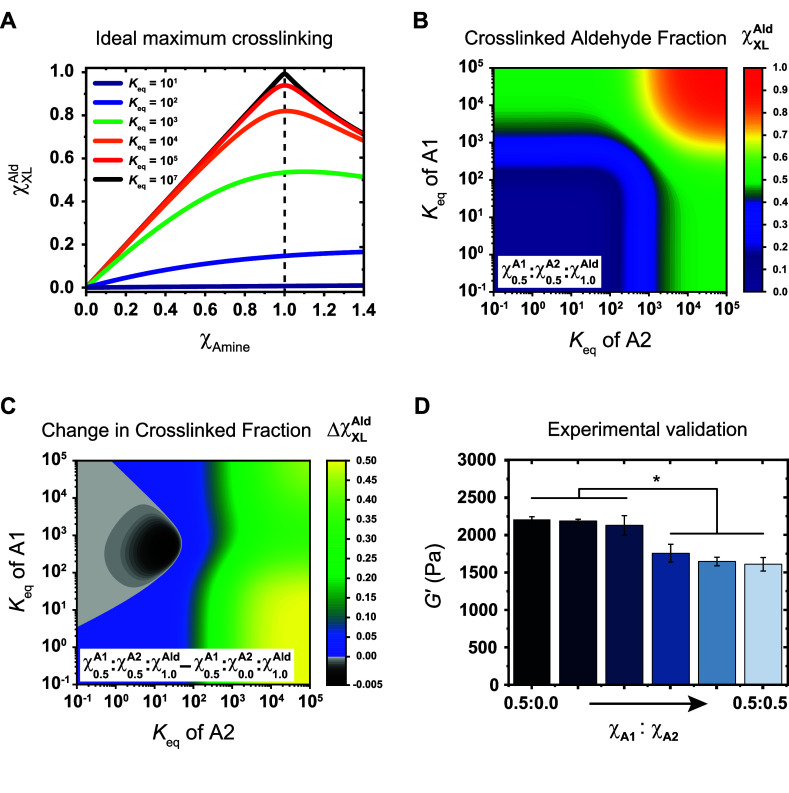
Theoretical determination and experimental validation of changes in cross-link concentration in competitively cross-linked systems reveals a counterintuitive regime where adding cross-linker reduces total number of cross-links. (A) Calculated evolution of the ideal maximum fraction of cross-linked aldehydes (χ_XL_^Ald^) as a function of the molar ratio of added amine (χ_Am_) for a series of different equilibrium constants (eq 1). (B) The calculated χ_XL_^Ald^ for a competitively cross-linked system containing 0.5 mol equiv. (w.r.t aldehydes) of two distinct amine cross-linkers, A1 and A2 (χ_0.5_^A1^:χ_0.5_^A2^:χ_1.0_^Ald^), as a function of their equilibrium constants (eqs 2 and 3). (C) The calculated difference in χ_XL_^Ald^ between a final system containing 0.5 mol equiv of two competitive amine cross-linkers (χ_0.5_^A1^:χ_0.5_^A2^:χ_1.0_^Ald^; see B) and an initial system containing 0.5 mol equiv of a single amine cross-linker (χ_0.5_^A1^:χ_0.0_^A2^:χ_1.0_^Ald^). The grayscale region indicates negative values corresponding to a decrease in χ_XL_^Ald^ upon the addition of A1 cross-linker. (D) The experimental validation of the grayscale region of negative values using bifunctional homologues of amines **5** and **7** (5* and 7*; See Materials and Methods) combined with aldehyde **2**, whose respective equilibrium constants are ≈10^3^ (**5** + **2**) and ≈10^2^ (**7** + **2**), and thus closest to the predicted negative values. Shear moduli were measured for a series of hydrogels starting with 0.5 equiv of 5*, and incrementally containing a further 0.1 equiv of 7*; the measured *G*′ values display the predicted softening behavior upon addition of amine 7*. *G*′ values are the mean ± standard deviation; *n* = 3. One-way ANOVA with Tukey’s multiple comparisons test, *P < 0.005.

Using an aldehyde concentration of 10.1 mM and an amine concentration ranging from 0–1.4 equiv with respect to the aldehydes, we generated the expected χ_XL_^Ald^ for *K*_eq_’s ranging from 10^1^ to 10^7^ (Figure 6A). Interestingly, we observe a high sensitivity to the value of *K*_eq_ in the regime between 10^2^ and 10^4^; at χ_Am_ = 1, χ_XL_^Ald^ drops from ≈0.8 at 10^4^ to ≈0.15 at 10^2^. This reveals a target range of *K*_eq_^’^s for imine formation, above which, the increase in the fraction of cross-links is small, and below which, negligible cross-linking will be observed. As we go beyond χ_Am_ = 1 (i.e., a molar excess of amine), the higher *K*_eq_ values are accompanied by a sharp drop in χ_XL_^Ald^ as the aldehydes are saturated and cross-links are replaced with singly bound amines. The magnitude of this decrease is reduced and the saturation point is shifted as *K*_eq_ decreases.

Next, we extended this simple model to competitive systems where two distinct amines displaying different *K*_eq_^’^s are present, and competing for the available aldehydes (Figure 6B). Importantly, the calculations are performed only up to χ_Am_ = 1, so we never reach an excess of amines, as this will lead to hydrogel softening via saturation.^28^ To achieve this, we employed a cubic solution to a system consisting of two competing ligands^74^ to determine the expected equilibrium concentration of remaining aldehyde for a given pair of amines and aldehyde at given initial concentrations and *K*_eq_^’^s (eq 2)
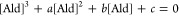
2where
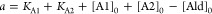

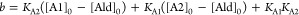
and
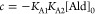


Here, *K*_A1_ and *K*_A2_ are the individual dissociation constants (inverse of the equilibrium constant) for each competing imine product, while [A1]_0_ and [A2]_0_ are the initial concentrations of each amine, [Ald] is the aldehyde concentration at equilibrium, and [Ald]_0_ is the initial aldehyde concentration. From this, we calculate the corresponding imine concentration for each competing amine according to eq 3
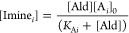
3

Here, [A_*i*_]_0_ is the initial concentration of either amine, while *K*_A*i*_ is the corresponding dissociation constant. We then determine χ_XL_^Ald^ according to eq 1 for each amine and add the obtained values together to get the total χ_XL_^Ald^ for the competitive system. Thus, we are able to calculate the total cross-linked aldehyde fraction—analogous with a hydrogels cross-link concentration (Figure 6B). Unsurprisingly, high equilibrium constants (>10^5^) resulted in near quantitative cross-linking. However, we could also see that the degree of cross-linking as a function of equilibrium constant had the same steep transition from 10^2^ < *K*_eq_ < 10^4^ that we observed for single amine calculations (Figure 6A). This transition region suggests that, contrary to some dynamic systems where a large range of equilibrium binding constants leads to a wide range of accessible mechanical properties, dynamic imine systems require fine control over a narrow *K*_eq_ region to effective tune their equilibrium bound fraction and resulting mechanical properties. We explore this idea further in a subsequent sections.

### Predicting Changes in Hydrogel Stiffness via Modeling of Competitive Cross-Linker Binding

With a model to describe changes in the equilibrium bound fraction of cross-linker (amines) as a function of *K*_eq_, we wanted to explore how we can use this approach to rationally engineer hydrogel stiffness, since *K*_eq_ ∝ *G*′ after cross-linking. In our previous work, we mixed amine cross-linkers in an inverted ratio (0:1–1:0 equiv A1:A2) and found a linear relationship between the cross-linker with the higher *K*_eq_ and the hydrogel’s shear modulus.^19^ It stands to reason that the equilibrium stiffness is largely determined by the stronger binding cross-linker in substoichiometric regimes (when entanglement is minimal). Here, we wanted to investigate whether adding a competitive cross-linker to a system can modulate the overall bound fraction. In other words, can mixed systems be used as a fine-tuning method for precisely inducing small changes in *G*′, and can we predict this using our simple model?

To set up a practical case for experimental comparison, we imagined a scenario in which a hydrogel system partially cross-linked with one amine evolves as a second amine cross-linker is titrated in (up to aldehyde saturation). In a simple case, we chose a system where χ_A1_ = 0.5 has a further χ_A2_ = 0.5 incrementally added to the system. We wanted to know how adding a competitive cross-linker modulates hydrogel stiffness as a function of the *K*_eq_’s of both competing amines (Figure 6C). The intuitive response is that adding more cross-linker to a hydrogel system adds more cross-links, and thus increases stiffness. This expected response holds true for competitors with higher *K*_eq_^’^s (>10^3^), but these calculations also reveal a counterintuitive region where the addition of a competitor decreases the overall cross-link concentration (grayscale region in Figure 6C). This finding predicts that in a dynamic hydrogel system cross-linked using A1 with a *K*_eq_ of ≈10^2^–10^3^, the addition of a competitor, A2, with *K*_eq_ < 10^2^ will result in a softening of the resulting hydrogel. Maximal softening is predicted at *K*_eq_(A1) = 4.5 × 10^2^ and *K*_eq_(A2) = 2 × 10^1^. In practice, we expect this region to be slightly shifted to higher *K*_eq_’s as values because our model will slightly overestimate the cross-linking efficiency; real hydrogel networks composed of telechelically functionalized macromers possess a fraction of nonideal intramolecular cross-links/loop-defects.

Consequently, to investigate this behavior experimentally, we selected **2** + **5** (*K*_eq_ = 1.3 ± 0.6 × 10^3^ L·mol^–1^) and **2** + **7** (1.0 ± 0.1 × 10^2^ L·mol^–1^) as these pairs possess *K*_eq_’s situated at the edge of the identified grayscale region. We prepared a series of 2 wt % hydrogels using aldehyde **2**, and bifunctional homologues of amines **5** and **7**, denoted as 5* and 7*, ranging from χ_A1_ = 0.5; χ_A2_ = 0.0 to χ_A1_ = 0.5; χ_A2_ = 0.5 in 0.1 equiv increments (Figure 6D). For this experiment, A1 = 5* while A2 = 7*. Remarkably, measurement of the resulting shear moduli of these hydrogels followed the predicted softening, with a decrease in *G*′ from 2200 Pa to 1600 Pa (a decrease of 27%). Again, the addition of cross-linker to the system decreased the stiffness of the hydrogel (without going over the saturation limit). To further link the observed difference in macroscopic mechanics to the molecular behavior predicted by our model, we quantified the imine concentrations in the initial and final formulations (0.5:0.0 and 0.5:0.0) presented in Figure 6D via ^1^H NMR (see Figure S12 and Table S3) using **2** + **5** and **2** + **7**. Comparing the 0.5:0.0 condition to the 0.5:0.5 condition, the total concentration of imine species increased slightly upon the addition of 0.5 equiv **7**. However, an increase in the absolute concentration of imines does not necessarily mean an increase in active cross-links, since an imine formed from 5* cannot participate in a cross-link with an imine formed from 7* and vice versa. When we consider each cross-linker separately by applying eq 1 to the individual amines (e.g., considering ([**5**′]/[**5**_0_])^2^ + ([**7**′]/[**7**_0_])^2^ instead of [**5**′] + [**7**′], where the prime indicates the imine form), we obtain a decrease in ideal imine cross-links of 12.5%, supporting the mechanical softening observed via rheometry.

Importantly, this emergent behavior is not accessible using covalent cross-linking, and may likely be present in other dynamically cross-linked systems. We also measured the stress relaxation behavior of these hydrogels (Figure S13), and noticed that the incremental addition of 7* resulted in a selective decrease in the early relaxation profile without impacting the slower overall relaxation end point. These findings demonstrate a new method for tuning material dynamics independent of traditional parameters (polymer content, composition, UV, pH, etc.),^7,75−79^ while highlighting the utility of precisely determined RECs for polymeric systems.

### Additional Considerations for Modeling Bulk Mechanical Properties from Molecular Rate and Equilibrium Constants

While the model developed in this work is tailored to examine trends and emergent behavior within a given system, taking advantage of the calculated concentrations of bound species to predict shear storage moduli is the next logical step. Notably, current models struggle to capture this molecular-macroscopic relationship in heterogeneous side chain functionalized dynamic macromeric systems. In this section, we contextualize and discuss this challenge.

Efforts to connect dynamic junction behavior to macroscopic material mechanics have explored different theoretical approaches including bond lifetime^91−93^ and transient network theory.^94−98^ More recently, a straightforward model to predict the shear storage modulus via the equilibrium constant of a dynamic reaction was developed by Parada and Parada.^99^ Anseth and colleagues also developed a modified phantom network model to account for the reversible nature of dynamic junctions in boronic aced ester hydrogels.^37^ However, these models, and previous efforts, rely on the (near) ideal behavior of telechelically functionalized multiarm networks. While promising, this limitation precludes the application of such models to the commonly used side-chain functionalized (bio)polymers, and entangled systems. To overcome these challenges, Spakowitz and colleagues developed Brachiation theory to account for both chain entanglement and longer-range hydrodynamic interactions.^12,100^ This unified approach produced excellent predictions of the rheological behavior of hydrogels across short and long time scales for a series of side-chain functionalized hyaluronic acid hydrogels. However, the underlying mathematics, despite leveraging predominantly experimentally accessible input parameters, remain challenging for nonexperts to implement and may hamper Brachiation theory’s widespread use.

**Figure 7:**
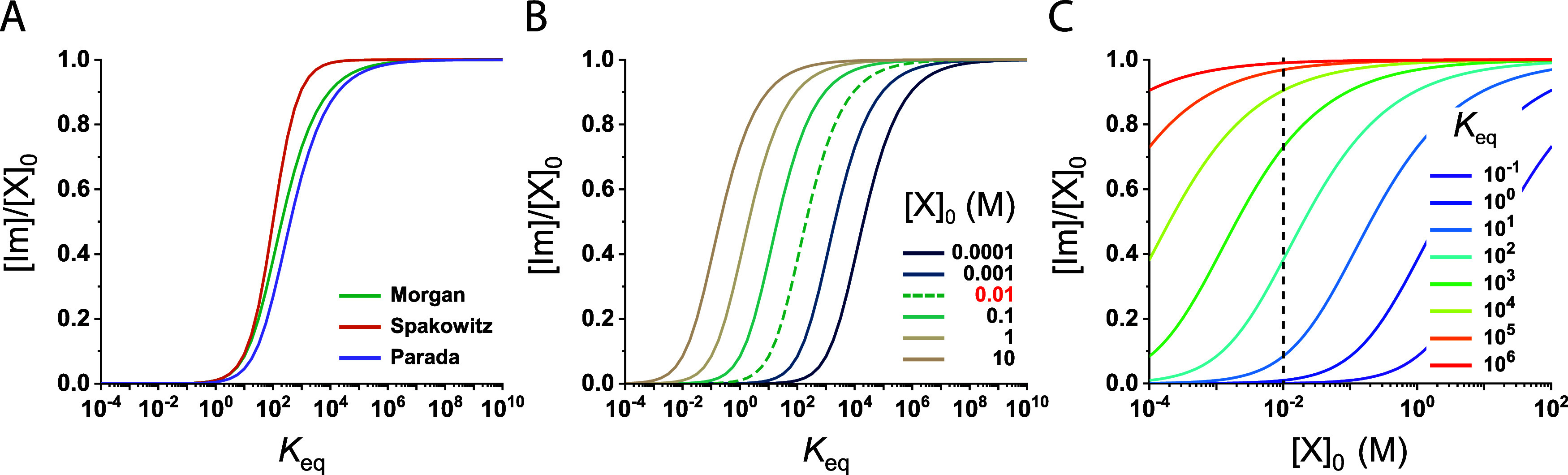
Comparison of expressions describing the equilibrium bound fraction of reactive groups and changes across different concentration regimes. (A) Comparison of the analytical solution to the bound fraction of reactive groups we used (Morgan)^74^ to the Bell model used by Parada and Zhao (Parada),^99^ and to the Poisson approach employed by Spakowitz and colleagues (Spakowitz).^100^ (B) The shape of the binding isotherm (Morgan) does not change shape but only shifts to higher or lower *K*_eq_ when the concentration regime changes. The same shift is observed in (Parada) and (Spakowitz) and shown in Figure S14. The concentration value highlighted in red accompanying the dotted line represents the concentration used for our study in Figure 6. (C) Plotting the bound fraction against the concentration regime for different *K*_eq_’s allows a direct comparison with the trend shown in Figure 6A, and facilitates the selection of an appropriate concentration regime of reactive groups for any system with a known *K*_eq_.

Collectively, the continued development of new models is building the foundation for further advancement. Interestingly, one element the dynamic models employed by Spakowitz and Parada both share with our model, is an expression describing the bound fraction of the chemical junctions at equilibrium. The approach to dynamic binding by Parada and Zhao is based on the Bell model of cell–cell or cell–substrate binding.^101^ Spakowitz and colleagues assume the reversible binding obeys a Poisson distribution and is independent of any mechanic forces at the binding sites.^100^ We use the analytical solution to a reversible bimolecular rate equation, extended to account for competitive binding.^74^ To explore the different treatments of equilibrium binding of these models we plotted the bound fraction at equilibrium ([Im]/[X]_0_) against *K*_eq_ for each of these models (Figure 7A). Here, [X]_0_ is the equimolar concentration of reactive partners (i.e., [X]_0_ = [Am]_0_ = [Ald]_0_ = 10.1 mM for our system), since not all models can accommodate nonequimolar conditions as derived. Similarly, we employ a single cross-linking function and equilibrium constant as currently, only our approach facilitates calculating mixed systems.

Comparing the different expressions employed to describe the bound fraction of reactive sites to our analytic approach, we see good agreement overall. The binding isotherm derived by Parada and Zhao is slightly shifter to higher *K*_eq_, while the approach by Spakowitz has a near-identical onset *K*_eq_ (threshold for bound fraction to start increasing) but a steeper rise and smaller *K*_eq_ for saturation of bound sites. Interestingly, all approaches predict the same narrow *K*_eq_ window of ≈3 decades in which significant control over the bound fraction of junctions can be influenced. This observation reinforces the idea that, for a given concentration regime, fine control over a narrow range of *K*_eq_’s will be more important the access to a wide range of *K*_eq_’s. Next, we looked at how this *K*_eq_ range changes depending on the reactive group concentration ([X]_0_, Figures 7B and S14). We chose a function concentration range to encapsulate commonly used systems. Here we observed a direct shift of the binding isotherm to higher/lower rangers for lower/higher concentrations in all approaches, indicating that the shape—and consequently the magnitude of the *K*_eq_ range—are independent of the reactive group concentration. Finally, we plot [Im]/[X]_0_ versus [X]_0_ (Figures 7C and S14) to enable direct comparisons with Figure 6A (dotted line) and facilitate the selection of a reactive group concentration that matches the *K*_eq_ for a known reaction, enabling robust design of hydrogel systems when RECs are known.

While the simple model we developed here can be used as an approximation for cross-link concentration, its strength lies in the prediction of changes in behavior within a given system. Particularly for any lab with an established system, the application of our model can enable the exploration of regimes and trends based on shifts in the molecular parameters governing junction formation. Additionally, our model facilitates the introduction of a competing cross-linking reaction to finely modulate the network properties and achieve robust control over mechanical properties within the effective *K*_eq_ range.

Dynamic binding models are continuing to develop, but a major remaining challenge for next generation predictive models relating molecular reaction parameters to macroscopic mechanics is the translation between a concentration of (dynamic) chemical junctions (reaction products such as imines) and the number of elastically active chain segments (*v*_e_), a common term used to relate *G*′ to network architecture. Similar quantities depending on the theoretical framework are also used. The true number of elastically active cross-links in a hydrogel network will depend not only the number of chemical junctions, but also physical junctions (entanglements). Additionally, not every chemical or physical junction will be elastically active at equilibrium, and defects such as loops and dangling reactive groups will further modify this value. From a fundamental perspective then, we can identify key variables including chain length, chain functionality, and the degree of chain entanglement. Chain length and functionality are readily characterized and often represented by describing the molecular weight between (chemical) cross-links.

However, entanglement is much more complex to describe accurately. First, it is important to know whether the macromer concentration fall into the dilute, semidilute, or concentrated regime to determine the amount of chain overlap. Indeed, Akagi and co-workers observed a transition in the predicted modulus of ideal tetra-PEG networks where below the critical overlap concentration the phantom network model was accurate, but above the critical concentration, the affine network model was more accurate.^80^ The degree of entanglement is further complicated by chain properties including the radius of gyration, persistence length, excluded volume (resistance to chain interpenetration) effects, and solvation among others.^81−83^ Several highly entangled hydrogel systems have been developed, highlighting the potential utility of known and controlled degrees of entanglement in soft material design.^84−86^ Accompanying efforts have developed new models to capture the impact of entanglements in hydrogel networks,^87,88^ yet these models remain complex to implement for nonexperts. Developing accessible generalized models able to encapsulate dynamic junctions alongside the impact of entanglements on soft material mechanics is an active area of research that we aim to contribute to in future work.

Currently, the most common classical elastic models to relate shear modulus to network topology describe affine and phantom networks. An affine model assumes all chain segments are elastically active and network junctions move proportionally to bulk deformation (which is representative of highly entangled networks);^89^ the phantom model assumes that junction positions can fluctuate to minimize the stress placed on network chains.^90^ Mathematically this difference is described by a correction term (1–2/*f*) for the effective elasticity of chain segments based on their functionality (*f*). The phantom model is more adapted to swollen hydrogel systems formed from dilute solution, as these swollen polymer networks are expected to have a lower frequency of polymer entanglements. Applying these models to our dynamic systems, we can use our measured *G*′ to calculate the concentration of elastically active chain segments (*v*_e_) under the conditions describing a 2 wt % oxidized alginate hydrogel with 0.5 equiv 5* cross-linker (Figure 6D, 0.5:0.0). We can then compare this value to a *v*_e_ predicted using knowledge of our macromers molecular weight, concentration and functionality. Both the affine and phantom network models predict a *v*_e_ of ≈0.9 mM, which is ≈5-fold lower than the 4.3 mM obtained using macromer-derived parameters (see Table S4). The high functionality of large side-chain functionalized polymers causes the correction term for effectively elastic chains in the phantom model to approach affine behavior. Consequently, the shear storage moduli predicted from chain concentration, composition and imine conversion using these models is approximately 5-fold higher than the measured value. Given the simplicity of the model and complex features of our dynamic, heterogeneous system, these differences between measured and predicted values are not surprising.

In future work, integration of dynamic binding models into network models relating junction concentration and architecture (e.g., RENT, affine theory, phantom theory, etc.),^80,89,102−105^ alongside additional consideration of entanglements and the impact of macromeric cross-linkers, will enable the development of robust predictive tools—specific to dynamic systems—to determine bulk material properties from known molecular parameters.

## Conclusion

In this work, we showed that slight chemical variation in amine nucleophiles leads to rate and equilibrium constants spanning 2–3 decades for a given aldehyde partner. Furthermore, differences in small molecule, natural polymer, and synthetic polymer aldehydes shifted the accessible range of *k*_1_, *k*_–1_ and *K*_eq_ up to 3 decades, underlining the importance of both reactants in determining their final molecular parameters, while highlighting the large overall range accessible even within a relatively small sample set (5 amines and 3 aldehydes). We established that our synthetic aliphatic aldehyde (**3**) is more reactive than a model small aldehyde molecule (**1**), while an oxidized natural polymer (**2**) is much less reactive. This enables the effective comparison of different polymeric systems to previous small molecule literature (notably in the field of chemical biology and bioconjugation) to provide a reasonable idea of the expected change in RECs when translated to a given polymeric system. Furthermore, we found that a simplified DFT model loosely fits experimental trends, but that more complex computational approaches will be necessary for future predictive applications.

Furthermore, we uncover an emergent phenomenon of softening of a dynamic hydrogel via competitive cross-linker addition. Extending our analysis of obtained rate and equilibrium constants to binding models, we first identified that the equilibrium cross-linked fraction is quite sensitive to *K*_eq_’s in the range of 10^2^–10^4^ L·mol^–1^. Subsequently, we were able to predict a counterintuitive regime where adding more cross-linker led to a decrease in overall cross-link concentration in an unsaturated system. Using our quantitatively determined *K*_eq_^’^s, we were then able to probe this regime and validated the predicted competitive softening regime. Notably, this method of modulating mechanical properties via binding dynamics is not accessible with traditional covalent hydrogels, and provides unique opportunities for tuning dynamically cross-linked systems in a variety of applications.

Finally, while our binding models are useful in their current form, future integration with network models will require the development of new network theories specifically adapted to dynamic cross-links. We anticipate that such developments will enable robust prediction of both network dynamics and bulk soft matter mechanics. Despite investigating structure–reactivity relationships in the context of dynamic covalent imine formation, the principles applied in this work should hold true for the general class of dynamic covalent chemistries and could be extended to noncovalent and supramolecular interactions. We anticipate that this work will be of use for the bottom-up design of any dynamically cross-linked polymeric system with quantified molecular parameters, with applications in soft matter engineering and recyclable materials.

## Materials and Methods

### Materials

Purified sodium alginate and the synthetic copolymer with pendant aldehydes (pSM-*co*-OMAm, **3**), were prepared as part of previous work. For details regarding their preparation, see our previous publication.^19,38^

All other materials were used as received unless otherwise specified. For the preparation of phosphate-buffered D_2_O, deuterium oxide (Sigma, 99.9 atom % D, D_2_O), potassium dideuterium phosphate (Sigma, 98 atom % D, KD_2_PO_4_), potassium phosphate tribasic (Sigma, ≥ 98%, K_3_PO_4_), and 3-(trimethylsilyl)-1-propanesulfonic acid-*d*_6_ sodium salt (Sigma, 98 atom % D, DSS-*d*_6_) were purchased from Sigma. For the oxidation of sodium alginate, we purchased sodium (meta)periodate (Sigma, ≥ 99.5%, NaIO_4_), ethylene glycol (Sigma, ≥ 99.5%), sodium chloride (Sigma, ≥ 99.0%), and SnakeSkin Dialysis Tubing (Thermo Fischer Scientific, MWCO = 3.5 kg·mol^–1^, 22 mm). Glutaraldehyde (Sigma, 50 wt % in H_2_O) and Schiff’s reagent (Sigma, 1.09034, Supelco) were purchased for the quantification of the degree of oxidation (DOx). For the small molecule kinetic studies, propionic acid hydrazide (PAH) (Sigma, ≥ 90%) was distilled after purchase while the synthesis of *N*-ethyl semicarbazide required ethyl isocyanate (sigma, ≥ 98%), hydrazine monohydrate (Sigma, 64–65% in H_2_O, ≥ 98%) and acetonitrile (VWR, catalogue# 1.00003.1000, MQ300). Other small molecules purchased for kinetic studies are 4-methyl-3-thiosemicarbazide (Sigma, ≥ 97%), propylamine (Sigma, ≥ 99%), ethylhydrazine hydrochloride (VWR, ≥ 95%), and *O*-ethylhydroxylamine hydrochloride (Sigma, ≥ 97%). Various stock solutions were prepared in Dulbecco’s PBS (Sigma, without MgCl_2_ or CaCl_2_). Adipic acid dihydrazide (Sigma, ≥ 98%), and *O*,*O*′-1,3-propanediylbishydroxylamine dihydrochloride (Sigma, 98%) were purchased as the homobifunctional homologues of PAH and *O*-ethylhydroxylamine hydrochloride, respectively.

### Preparation of Phosphate Buffered D_2_O for NMR Analyses

To a fresh 100 g bottle of D_2_O we added 381 mg of K_3_PO_4_, 463 mg of KD_2_PO_4_, and 100 μL of 100 mM DSS-*d*_6_ stock to yield a final phosphate concentration of 56.9 mM and DSS-*d*_6_ concentration of 0.11 mM. The pH was measured to be 7.42, and is expected to closely match the pD.^106^ Unless otherwise specified, all D_2_O NMR spectra were measured in this phosphate buffered D_2_O.

### Preparation of Oxidized Alginate (**2**)

In a 150 mL glass beaker, 1.0 g purified alginate (1.0 equiv, 5.05 mmol of uronic acid units) was dissolved in 98 mL Milli-Q (1.0 wt %) water while sodium periodate (124.2 mg, 0.581 mmol, 0.115 equiv w.r.t. moles of uronic acid units, theoretical final DOx 11.5%) was dissolved in 2.0 mL Milli-Q water. The reaction flask was then covered in aluminum foil to protect it from light, the sodium periodate solution was added to the purified alginate solution, and the reaction was allowed to proceed for 17.5 h. The reaction was quenched by adding ethylene glycol (35.6 μL, 0.127 equiv w.r.t. moles of uronic acid units) directly to the reaction mixture and leaving it for an hour. The crude reaction mixture was subsequently transferred to dialysis tubing and dialyzed sequentially against 100 mM, 50 mM, 25 mM 12.5 mM and 2 × 0 mM NaCl solutions in distilled water (dH_2_O) (changed every morning and evening) before being frozen and lyophilized. A typical yield was 80–84%, with *M*_n_ = 130 kg·mol^–1^ and *D̵* = 2.2.

### Quantification of the DOx of Sodium Alginate (**2**)

The DOx of oxidized alginate (**2**) is defined here as the percent of uronic acid units that are oxidized; each oxidized uronic acid unit generates two aldehyde functions (i.e., the number of moles of aldehyde is twice the number of moles of oxidized uronic acid units when preparing subsequent stock solutions). The DOx was determined using the Schiff test.^107−110^ First, a standard series of 500 μL glutaraldehyde solutions (20–0.5 mM) was prepared in dH_2_O. Then 2500 μL of Schiff’s reagent was added at staggered time points to each sample, and exactly 40 min later, a 100 μL aliquot was diluted to 1000 μL with dH_2_O in a quartz cuvette with a path length of 1 cm; the absorbance was recorded from 400–700 nm at 25 °C with a scan rate of 300 nm·min^–1^ and averaging time of 0.1 s. The final aldehyde concentrations for the standard series were 333, 167, 83, 33, and 8 μM. The slope of a linear fit gives the molar absorptivity coefficient (1510 L·mol·cm^–1^). A 1 wt % solution of oxidized alginate (**2**) was then prepared to determine its aldehyde content, and corresponding DOx, which was found to be 10% (Figure S15).

### Distillation of PAH (**7**)

Using short-path vacuum distillation apparatus connected to a Schlenk line, 400 μL PAH (≥ 90%) was loaded into a 5 mL round-bottomed flask. The pressure was reduced to <1 mbar (varied from 0.2–0.9 mbar) and the flask was heated at 85 °C in an oil bath. Glass wool, aluminum foil and a heat gun were used to keep the glass joint and path to the condenser at a comparable temperature. After 2.5 h the distillation began to noticeably slow down and the mother liquor had significantly yellowed. The distillate (colorless liquid) was stored under N_2_, allowed to cool, then further cooled in the fridge to yield a white crystalline solid (213 mg, 53%). ^1^H NMR analysis indicated a purity of >99%. ^1^H NMR (700 MHz, D_2_O, δ in ppm): δ 1.10 (t, 3H, *J* = 7.7 Hz), 2.21 (q, 2H, *J* = 7.6 Hz).

### Synthesis of N-Ethyl Semicarbazide (**8**)

A 2 mL acetonitrile solution of ethyl isocyanate (200 mg, 2.8 mmol) was added dropwise to a 3 mL acetonitrile solution of hydrazine monohydrate (867 mg hydrazine, 11.3 mmol, 4 equiv) in a 25 mL brown glass round-bottomed flask precooled using an ice-salt bath. The reaction was allowed to proceed overnight without replacing the ice-salt bath. Acetonitrile was removed under reduced pressure at ambient temperature, and then the temperature was then increased to 30 °C for 4 h to remove residual water. *N*-ethyl semicarbazide was recovered as a colorless oil, which, upon cooling to 4 °C, became an opaque waxy solid (227 mg, 78%) and was stored at 4 °C under N_2_. ^1^H NMR (700 MHz, DMSO-*d*_6_, δ in ppm): δ_H_ 1.00 (t, 3H, *J* = 7.1 Hz, H-a), 3.04 (dq, 2H, *J* = 7.2, 5.9 Hz, H-b), 4.06 (br, 2H, H-f), 6.28 (br, 1H,H-c), 6.86 (s, 1H, H-e). ^13^C (176 MHz, DMSO-*d*_6_, δ in ppm): δ_C_ 15.7 (s, C-a), 33.5 (s, C-b), 160.0 (s, C-d). ^1^H and ^13^C spectra, along with 2D ^1^H–^15^N HSQC and ^1^H–^15^N HMBC can be found in Figures S16, S17, S18, and S19, respectively.

### Density Functional Theory Computational Details and Methodology

Calculations in this study were performed at the B3LYP/6-31+G(d,p) level of theory at standard temperature (298.15 K) and pressure (1 atm) using the Gaussian 09 software package. Geometries were optimized in water as a nonexplicit solvent using the integral equation formalism polarizable continuum model by screening a series of conformational isomers around double bonds. Frequency calculations were performed to determine Gibbs free energies for each structure, and validated as true minima by the absence of any negative frequencies. The Gibbs free energies for reaction step were calculated according to eq 4.
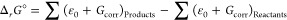
4

### Determination of Molar Absorptivity Coefficients

Aqueous stock solutions of amines **5**–**9** were prepared at 20 mM while stock solutions of aldehydes **1**–**3** were prepared at 10 mM. Samples containing a constant aldehyde concentration of 500 μM, and amine concentrations of 167, 333, and 500 μM were prepared in phosphate buffered D_2_O. After at least 2 h, each sample was measured by both UV–vis and ^1^H NMR spectroscopy. These measurements were carried out within 5 min of each other for each sample. UV–vis spectroscopic measurements were performed on an Agilent Cary 60 UV–vis spectrophotometer equipped with a Peltier temperature controller. Spectra were acquired from 300–200 nm with a scan rate of 396 nm·min^–1^ and steps of 0.33 nm at 20 °C. ^1^H NMR spectra were recorded on a Bruker Avance III HD 700 MHz spectrometer equipped with a cryogenically cooled three-channel TCI probe, and analyzed with the TopSpin 4.0 software (Bruker, Germany). The internal DSS-*d*_6_ enables quantification of the imine concentration via NMR. Subsequently, a plot of absorbance (from UV–vis) against imine concentration yields the molar absorptivity coefficient as the slope of a linear fit. These plots and resulting molar absorptivity coefficients can be found in Table S1 and Figure S4. Due to the detection limits of NMR, our concentration for the determined molar absorptivity coefficients did not go below the μM range.

### Measurement of **2** + **5** and **2** + **7** Reaction Kinetics via ^1^H NMR

Stock solutions of **2**, **5**, and **7** were prepared in phosphate buffered D_2_O at appropriate concentration for dilution to the final concentrations given in Table 2. A sample containing only **2** was used to shim the NMR probe prior to the kinetic measurements to optimize sensitivity. **2** was then mixed with either **5** or **7** to achieve the 5 or 24 mM used for acquisition and loaded in the NMR. After 5 min (time to load and set up) acquisition was started. Spectra were acquired continuously and stored in a pseudo 2D acquisition file where the second dimension stores the chosen average of 16, 32, or 64 scans. Exporting slices at each row of the second dimension yields a series of ^1^H NMR spectra over time. These files were then processed using MATLAB to baseline correct, reference to 0 ppm using the DSS-*d*_6_ internal standard signal, integrate predetermined regions corresponding to the internal standard and imine products, and convert the final integrals to concentration. In the case of **2** + **5** this was the shifted terminal methyl signal at 1.19–1.30 ppm, while for **2** + **7**, this was the shifted terminal methyl signal at 1.13–1.18 ppm. The resulting concentration vs time data was plotted as the consumption of **2** or **5** over time and fitted to the kinetic model described by eq 5 (vide infra) to extract rate and equilibrium constants.

### Measurement of Reaction Kinetics via UV–vis Spectroscopy

UV–vis spectroscopic measurements were performed on an Agilent Cary 60 UV–vis spectrophotometer equipped with a Peltier temperature controller. Spectra were acquired every 30 s for 7200 s (2 h) from 300–200 nm with a scan rate of 396 nm·min^–1^ and steps of 0.33 nm at 20 °C. For each measurement, a quartz cuvette (Hellma Analytics, 114F-10–40, light path = 10 mm × 4 mm) was first cleaned with water and ethanol, and then dried with compressed air. The absorbance signal was zeroed against air and a baseline of PBS was recorded. Stock solutions of aldehydes **1**–**3** (10 mM) and amines **4**–**9** (20 mM) were prepared in PBS. In the case of aldehydes **2** and **3**, the aldehyde concentration was calculated according to , where *m* is the mass of macromer added, *M*_Unit_ is the average monomer molecular weight, *D*_f_ is the degree of aldehyde functionalization [10% for **2** (see method above); 29% for **3** via ^1^H NMR as we previously reported^38^, and *V* is the volume. The aldehyde was added to the PBS used for the baseline measurement and thoroughly mixed by pipetting 200 μL up and down 15–25 times. The sample was allowed to equilibrate thermally for 10 min prior to the addition of the amine partner. The reaction mixture was homogenized by pipetting and data acquisition was started exactly 25 s after amine addition. Kinetic data (Figure 3) are reported from 210 nm as this corresponds to the solvent cutoff wavelength for PBS.

### Fitting of UV–Vis Data to a Second Order Bimolecular Reversible Rate Equation to Obtain Rate and Equilibrium Constants

Kinetic data were processed by subtracting the first spectrum from all other spectra to correct for background signal from 1–9 prior to imine formation. We thus obtain the change in absorbance over time, which is converted (using ε) to a plot of concentration over time for the reacting amine (not imine), as the model fits the consumption of the amine over time. The forward (*k*_1_) and reverse (*k*_–1_) rate constants for imine formation (amine consumption) were determined by fitting the kinetic data to the solution for an equimolar, bimolecular, reversible reaction following second order kinetics developed by Dirksen et al.^61^ This equation (eq 5) is reproduced below for convenience, and describes the disappearance of the amine in time; where *x*_0_ is the initial amine concentration and *x*(*t*) is the remaining amine concentration as some point in time (*t*). Fitting was done by programming eq 5 into Origin 2018 (OriginLab).
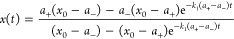
5where
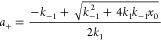
and
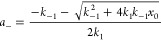


### Preparation of Hydrogels to Validate the Predicted Softening Regime

Aldehyde **2** and amines **5** and **7** were selected to most closely match the predicted competitive softening regime. To investigate bulk hydrogel properties we used homobifunctional homologues to enable cross-linking: In place of **5** we used *O*,*O*′-1,3-propanediylbishydroxylamine (5*), while in place of **7**, we used adipic dihydrazide (7*). Stock solutions of **2** (4 wt % in PBS), 5* (100 mM in PBS), and 7* (100 mM in PBS), adjusting to pH = 7.4 with dilute NaOH where necessary (as 7* comes as the hydrochloride salt) were prepared. Hydrogels ranging from χ_0.5_^A1^:χ_0.0_^A2^:χ_1.0_^Ald^ to χ_0.5_^A1^:χ_0.5_^A2^:χ_1.0_^Ald^ in 0.1 equiv increments were prepared in PBS, where A1 = 5* and A2 = 7*. The molar ratio of each amine to aldehyde was determined as the ratio of the concentrations of amine functions to aldehyde functions (accounting for the bifunctional nature of the cross-linkers). The final concentration of **2** in hydrogels was 2 wt % ([aldehyde] = 10 mM), with 5 mM of an amine function for 0.5 equiv (which also corresponds to 2.5 mM 5* or 7* for example as they are homobifunctional molecules).

### Rheometry of Hydrogels to Validate the Predicted Softening Regime

Shear moduli were measured on a DHR-2 rheometer from TA Instruments equipped with a Peltier plate and 8 mm parallel plate geometry. Hydrogels were formed in advance in silicon molds, loaded into the rheometer, trimmed, and then the gap was lowered to achieve a normal force of 0.1 N. The final gap size across different samples varied from ≈880 to 980 μm. To assess the shear moduli, time sweeps were recorded for 360 s at 1% strain, 1 rad·s^–1^ at 20 °C. The *G*′ value for each sample was taken to be the final value at the end of 360 s. Replicates (*n* = 3) for each sample were then used to determine the mean ± standard deviation for each formulation.

The stress relaxation profiles of the hydrogels were measured using a 20 mm cone-plate upper geometry with an angle of 2.002°. After loading 84 μL of a precursor solution into the rheometer, gelation was followed in situ with an oscillatory time sweep (γ = 1%, ω = 10 rad·s^–1^, *T* = 20 °C) for 2.5 h to allow a stable plateau to be reached. Subsequently, a 20% strain was applied to the sample and then stress relaxation profile was recorded over 15.5 h, still at 20 °C. Throughout these measurements, the rheometer was equipped with a solvent trap to prevent any solvent evaporation. This was verified by inspection of the sample after the measurement to ensure no sample shrinkage had occurred.

## Data Analysis and Statistics

Statistical analyses were performed using Origin 2018 SR1. The exact statistical test is specified in figure legends and the relevant methods sections. Fitting of kinetic data was performed in Origin 2018 SR1 using the nonlinear curve fitting tool. Unless otherwise stated, error bars represent the mean ± standard deviation.

## Data Availability

The data that support the findings of this study are openly available in DataverseNL at https://doi.org/10.34894/BQNQJX.

## Supporting Information Available

Additional tables and figures for molar absorptivity, kinetic, UV–vis, NMR and rheological data alongside details of the model used relate the equilibrium constant to the cross-linked fraction of aldehydes (PDF)
