# Supplementary material for: Structure–Reactivity Relationships in a Small Library of Imine-Type Dynamic Covalent Materials: Determination of Rate and Equilibrium Constants Enables Model Prediction and Validation of a Unique Mechanical Softening in Dynamic Hydrogels
Source: J Am Chem Soc. 2024 Oct 1;146(40):27499–516. doi: 10.1021/jacs.4c08099 (PMC11467966; doi:10.1021/jacs.4c08099)
Supplement: Supplementary file 1 — ja4c08099_si_001.pdf [file ja4c08099_si_001.pdf]

Supporting information

Structure-reactivity relationships in a small library of imine-type dynamic covalent materials: Determination of rate and equilibrium constants enables model prediction and validation of a unique mechanical softening in dynamic hydrogels

Francis L. C. Morgan,[1,2] Ivo A. O. Beeren,[1,2] Jurica Bauer,[2] Lorenzo Moroni,[2] Matthew B. Baker[1,2]*

[1]Department of Instructive Biomaterials Engineering, MERLN Institute for Technology-Inspired Regenerative Medicine, Maastricht University, 6229 ER, Maastricht, The Netherlands

[2]Department of Complex tissue Regeneration, MERLN Institute for Technology-Inspired Regenerative Medicine, Maastricht University, 6229 ER, Maastricht, The Netherlands

*Corresponding author. Email: m.baker@maastrichtuniversity.nl

TABLE OF CONTENTS

Supporting Tables

Table S1 Summary of molar absorptivity coefficient values via NMR & UV-Vis.

Table S2 Summary of fitting **3+8**, **3+9**, and **2+9** to irreversible second order kinetic behavior.

Table S3 Quantification of imine concentration via ^{1}H NMR (700 MHz, D_2O, DSS-d_6).

Table S4 Prediction of elastically active chain segments using classical affine and phantom network models.

Supporting Figures

Figure S1 ^{1}H NMR (700 MHz, D_2O, DSS-d_6) spectra of tested aliphatic aldehydes.

Figure S2 Overall and simplified reaction coordinates for imine formation.

Figure S3 Plots of calculated natural charge and nucleophilicity against each other and $\Delta G_1^*/RT$.

Figure S4 Effect of salt type and concentration on **2**+**5** UV-Vis rate constant.

Figure S5 Determination of molar absorptivity coefficients via NMR & UV-Vis.

Figure S6 ^{1}H NMR (700 MHz, D_2O, DSS-d_6) spectrum of propylamine (**4**) and pSM-*co*-OMAm (**3**) after 16 h.

Figure S7 Possible 8-membered ring stabilization of the transition state via H-bonding.

Figure S8 ^{1}H NMR (700 MHz, D_2O, DSS-d_6) spectra of aldehydes **1**–**3**.

Figure S9 Second order kinetic fits of 1/[Amine] vs time for **3+8**, **3+9**, and **2+9**.

Figure S10 Comparison of the mean rate and equilibrium constants all aldehyde-amine pairs grouped by amine.

Figure S11 Rate-equilibrium free energy relationship plot of Log(k/k_0) vs Log(K/K_0).

Figure S12 Quantification of imine concentration via ^{1}H NMR (700 MHz, D_2O, DSS-d_6).

Figure S13 Stress relaxation behavior of mixed hydrogels of **2**+**5***+**7***.

Figure S14 Model plots comparing binding isotherms and bound fractions *versus* concentration using the approaches of Spakowitz and colleagues and Parada and colleagues.

Figure S15 Quantification of the degree of oxidation (DOx) of sodium alginate.

Figure S16 ^{1}H NMR (700 MHz, DMSO-d_6) spectrum of **9**.

Figure S17 ^{13}C NMR (176 MHz, DMSO-d_6) spectrum of **9**.

Figure S18 ^{1}H–^{15}N HSQC NMR (700 MHz, DMSO-d_6) spectrum of **9**.

Figure S19 ^{1}H–^{15}N HMBC NMR (700 MHz, DMSO-d_6) spectrum of **9**.

Model for maximum ideal crosslinked aldehyde fraction as a function of equilibrium constant and mole fraction of added amine.

Supporting Tables

Table S1. Summary of molar absorptivity coefficients obtained via NMR and UV-Vis for each amine-aldehyde pair in this study.

Aldehyde-Amine Pair	λ_{max} (nm)	ε (L·mol⁻¹·s⁻¹)	δ_{Imines} (ppm)[a]
1+5	220	2900	6.69, 7.44
1+6	235	4750	6.56, 7.18
1+7	240	11100	6.79, 7.31, 7.46
1+8	230	10200	6.50, 6.79, 7.22
1+9	265	20500	6.61, 7.37
2+5	220	950[b]	n.d.[c]
2+6	235	2100	n.d.[d]
2+7	240	1870[b]	n.d.[e]
2+8	230	8700	6.63, 6.93, 7.30
2+9	265	7200	n.d.[f]
3+5	220	2200	6.91, 7.49
3+6	235	4200	6.71, 7.24
3+7	240	8600	6.96, 7.40, 7.54
3+8	230	9700	6.67, 6.94, 7.26
3+9	265	14200	6.79, 7.42

[a] The integrals of these peaks, in combination with the DSS-d_6 I.S. were used to determine imine concentration.

[b] Pairs 2+5 and 2+7 report the average value obtained from two experiments.

[c] The imine peaks were broad, and too close to the baseline noise level to be reliable. The shifted methyl triplet of the imine product at 1.24 ppm was used instead.

[d] The imine peaks were broad, and too close to the baseline noise level to be reliable. The shifted methyl triplet of the imine product at 1.36 ppm was used instead.

[e] The imine peaks were broad, and too close to the baseline noise level to be reliable. The shifted methyl triplet of the imine product at 1.14 ppm was used instead.

[f] The imine peaks were broad, and too close to the baseline noise level to be reliable. The shifted methyl singlet of the imine product at 3.07 ppm was used instead.

Table S2. Summary of fitting 3+8, 3+9, and 2+9 to irreversible second-order kinetic behavior.

Imine	Replicates	k_1 (10^{-2}·L·mol⁻¹·s⁻¹)	1/[Amine]$_0$ (L·mol⁻¹)	Mean r^2
3+8	4	403 ± 22	9500 ± 185	0.9997
3+9	4	75.5 ± 3.8	19905 ± 12	0.9997
2+9	4	5.9 ± 0.0(3)	19980 ± 13	0.9945

Table S3. Summary of integration values and corresponding concentrations determined from Figure S12.

Formulation	Signal	ppm	Integration (I_x)	Concentration (C_x, mM)[a]	N_x
0.5:0.0	Imines	6.60–8.00	2.849	2.82	1
0.5:0.0	**5** Methyl (unbound)	1.15–1.19[b]	4.386	1.45	3
0.5:0.0	**5** Methyl (imine)	1.20–1.30	12.531	4.14	3
0.5:0.5	Imines	6.60–8.00	3.165	3.13	1
0.5:0.5	**5** Methyl (unbound)	1.15–1.19[b]	5.475	1.80	3
0.5:0.5	**5** Methyl (imine)	1.20–1.30	12.158	4.01	3
0.5:0.5	**7** Methylene (unbound)	2.18–2.25	11.410	5.65	2
0.5:0.5	**7** Methylene (imine)	2.27–2.35	0.796	0.39	2

[a] The concentration was determined according to $C_x = I_x/I_{cal} * N_{cal}/N_x * C_{cal}$, where x represent the signal whose concentration we are determining and 'cal' represents the internal DSS-d_6 standard, I is the integration values, and N is the number of protons giving rise to the signal. N_{cal} is 9, I_{cal} is set to 1.000, and C_{cal} is 0.11 mM.

[b] The integration range for unbound **5** has been slightly truncated to avoid contributions from the adjacent **7** Methyl (imine) signal.

Table S4. Prediction of elastically active chain segments using classical affine and phantom network models and comparison with our calculated chemical junction concentrations.

Model	Equation	$G' \rightarrow v_e$[a] (mM)	$v_e = \rho/M_c$ (mM)[b]	$v_e \rightarrow G'$ (kPa)[c]
Ideal affine network	$G' = v_e k_B T$	Ve = 0,90	4.38	10.5
Ideal phantom network	$G' = v_e k_B T(1 - 2/f)$	Ve = 0.87[d]	4.38	10.5

[a] Values determined using the shear storage modulus from **Figure 6A** with 0.5 equiv of **5*** (2200 Pa), where k_b is the Boltzmann constant ($1.38 \cdot 10^{-23}$ J·K^{-1}), and T is the temperature in Kelvin. The obtained value is then divided by Avogadro's number to yield a molar concentration.

[b] The theoretical v_e is determined as the ratio of polymer chain concentration (ρ in g·dm^{-3}; 2 wt% hydrogel) to the average molecular weight between crosslinks (M_c). Here, M_c is determined by $M_c = (M_n/f) * (1/(0.5 * \text{Conv}))$, where 'Conv' is the equilibrium conversion of **5*** to an imine at 0.5 equiv, and the 0.5 scaling factor is because 0.5 equiv **5*** were used.

[c] The shear storage modulus is predicted here using the theoretical v_e determined as described '[b]'.

[d] The functionality of an oxidized alginate chain with M_n = 114 kg·mol^{-1}, an average monomer unit molar mass of 197.9 g·mol^{-1}, and a 10% degree of functionalization is ≈ 57.

Supporting Figures

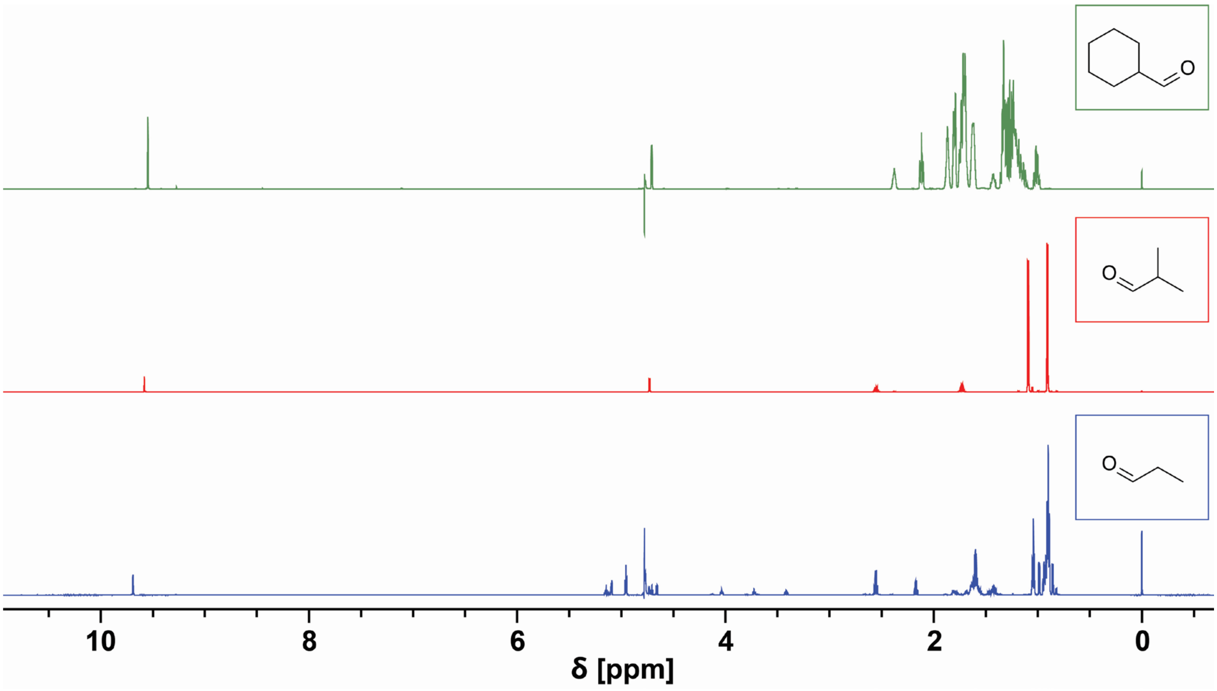

Figure S1. ^{1}H NMR (700 MHz, D_2O, DSS-d_6) spectra of tested aliphatic aldehydes. Cyclohexanecarboxaldehyde (top, green) has complex coupling patterns and is sterically constrained, while propionaldehyde (bottom, blue) has complex secondary forms. In contrast, isobutyraldehyde has a clean, simple structure and no secondary forms. The negative peak at 4.7 ppm in cyclohexanecarboxaldehyde is from the water suppression integrated into the pulse sequence.

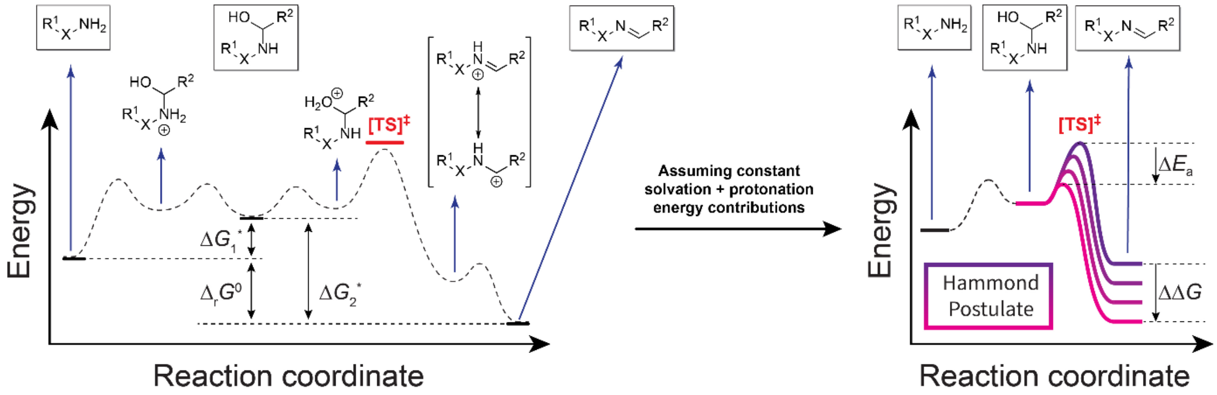

Figure S2. Overall and simplified reaction coordinate-energy diagram for imine formation. According to Scheme 1, we have estimated the overall relative energy changes over the course of imine formation. Dotted lines are intended as representative shifts and are not empirically determined. The chosen structure for DFT modeling and their respective Gibbs energies obtained are indicated by thick solid lines and labelled vertical arrows respectively. By applying the Hammond postulate, and assuming solvation and protonation parity across the series, we obtain a simplified reaction coordinate-energy diagram to use as an approximate model of our system.

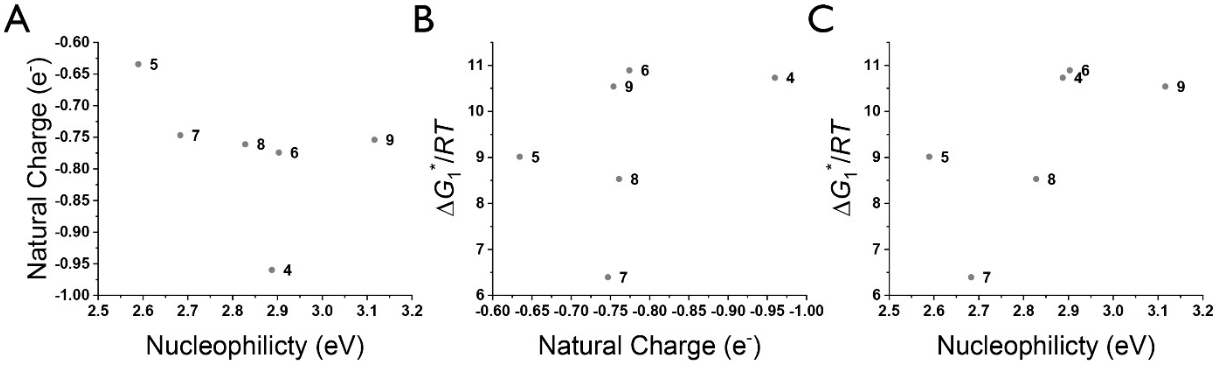

Figure S3. (A) A plot of the natural charge vs nucleophilicity reveals a weak linear correlation between the nucleophilicity of the labelled amine and the magnitude of the natural charge on the terminal nitrogen. This trend is consistent with the rationale typically used in organic chemical reactions that stronger nucleophiles react faster under equivalent conditions. Similarly, plots of each of the natural charge (B) and nucleophilicity (C) vs the calculated Gibbs free energy associated with the nucleophilic attack step of imine formation also show weak linear correlations where increasing either the charge or nucleophilicity tends towards a faster formation of the tetrahedral intermediate.

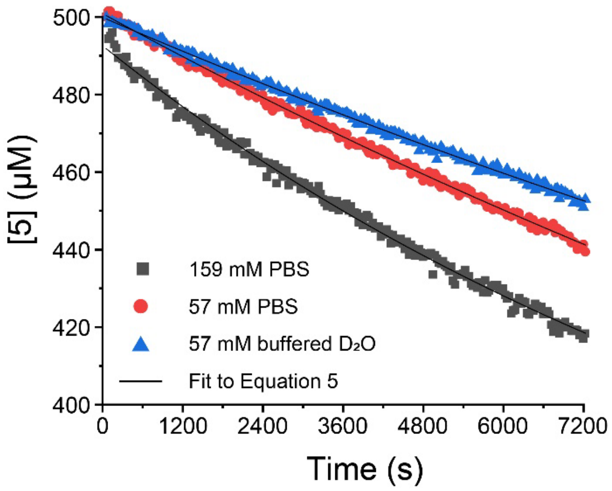

Figure S4. Comparison of the effect of salinity on imine formation with **2+5**. Decreasing the salinity from 159 mM PBS to 57 mM PBS decreases the rate of imine formation (k_1) from $5.8 \cdot 10^{-2}$ L · mol^{-1} · s^{-1} (grey) to $3.7 \cdot 10^{-2}$ L · mol^{-1} · s^{-1} (red). Maintaining the salt concentration of 57 mM but switching to the deuterated phosphate buffer used for NMR studies further decreases the k_1 to $2.9 \cdot 10^{-2}$ L · mol^{-1} · s^{-1} (blue).

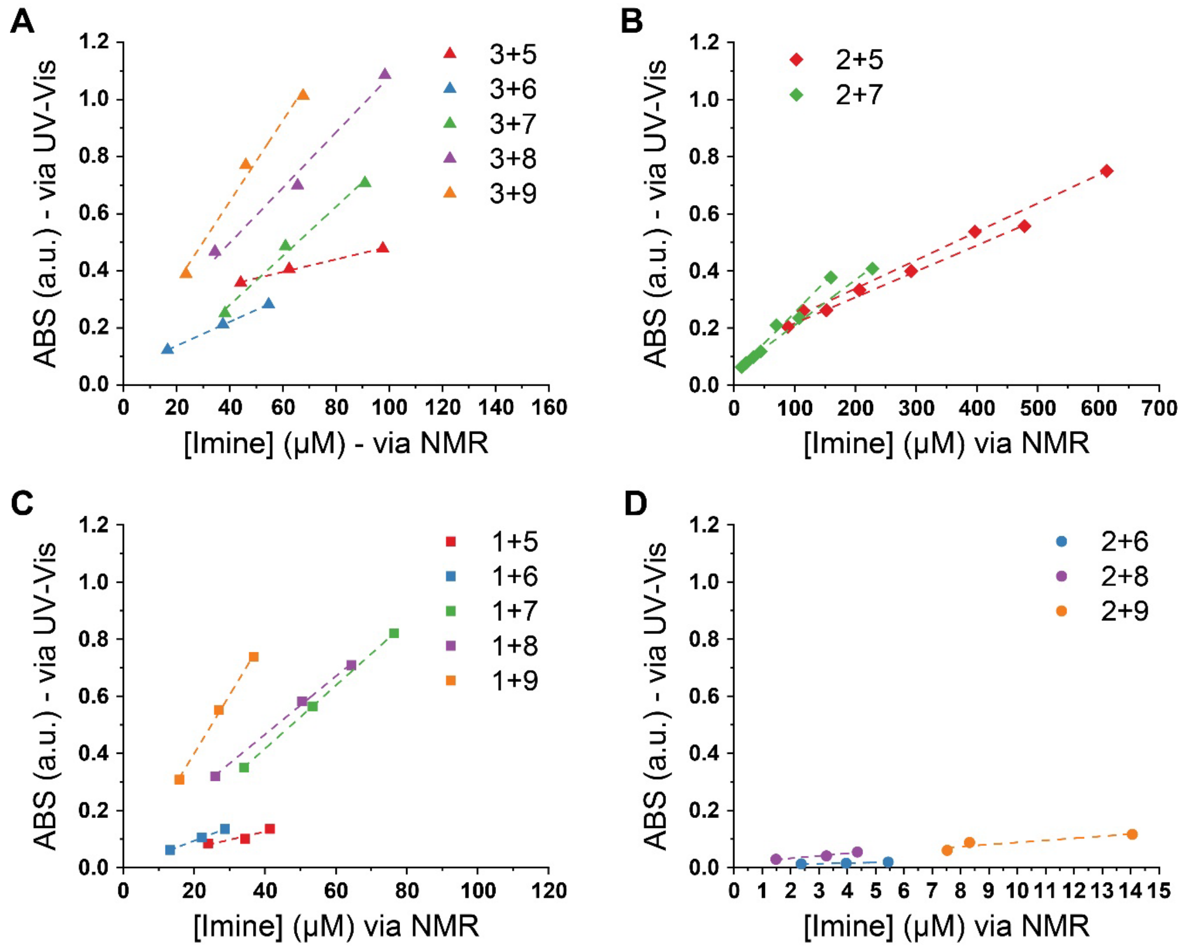

Figure S5. Plots of concentration (via NMR) vs absorbance (UV-Vis) for the determination of molar absorptivity coefficients for each amine-aldehyde pair. The determination of **2+5** and **2+7** was repeated once to test the reproducibility of our method, which was found to reproducible. The molar absorptivity coefficient is given by a linear fit according to the Beer-Lambert law ($A = \varepsilon \cdot c \cdot l$), where the slope corresponds to ε (path length l = 1 cm). The integrals used to determine concentration via NMR are listed in **Table S1**.

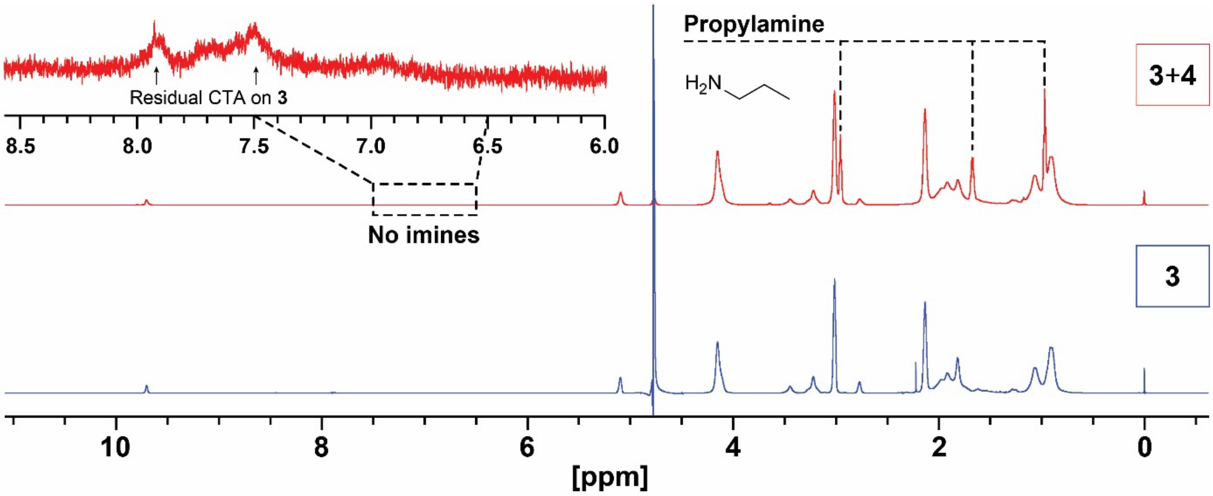

Figure S6. ^{1}H NMR (700 MHz, D_2O, DSS-d_6) spectra of propylamine (**4**) and pSM-*co*-OMAm (**3**) after 16 h compared to **3** in the absence of **4**. We observe no imine peaks in the spectrum; the noisy signals highlighted in the inset are from the chain transfer agent (CTA) used during the copolymerization of **3**.

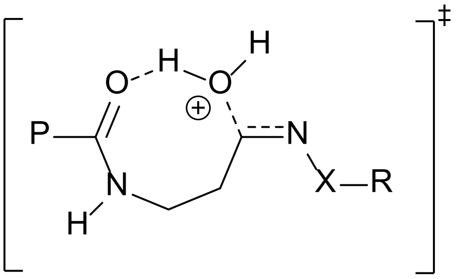

Figure S7. Possible 8-membered ring stabilization of the transition state via H-bonding. Here, P is the polymer (**3**) backbone while the possibilities for X and R are indicated in **Figure 2**.

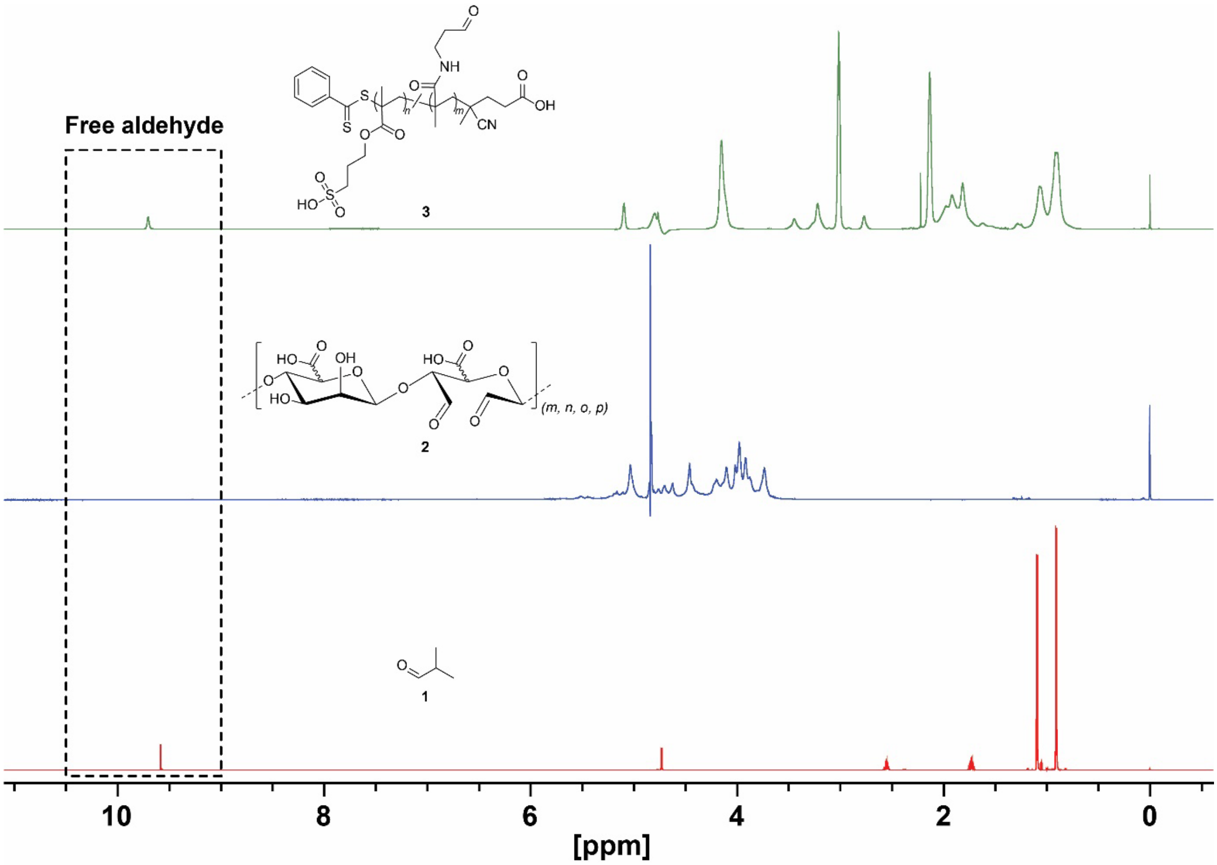

Figure S8. ^{1}H NMR (700 MHz, D_2O, DSS-d_6) spectra of aldehydes **1**–**3**. Oxidized alginate (**2**) has no signal for free aldehydes.

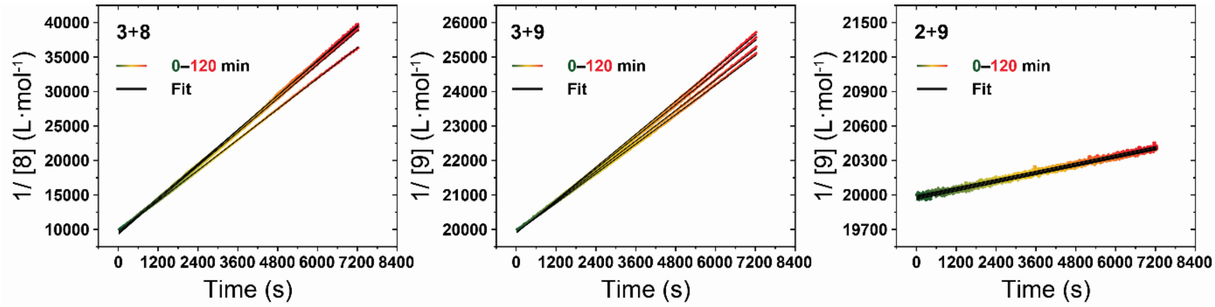

Figure S9. Irreversible second-order linear kinetic fits of 1/[Amine] vs time for **3+8**, **3+9**, and **2+9**. Fitting results are summarized in **Table S2**.

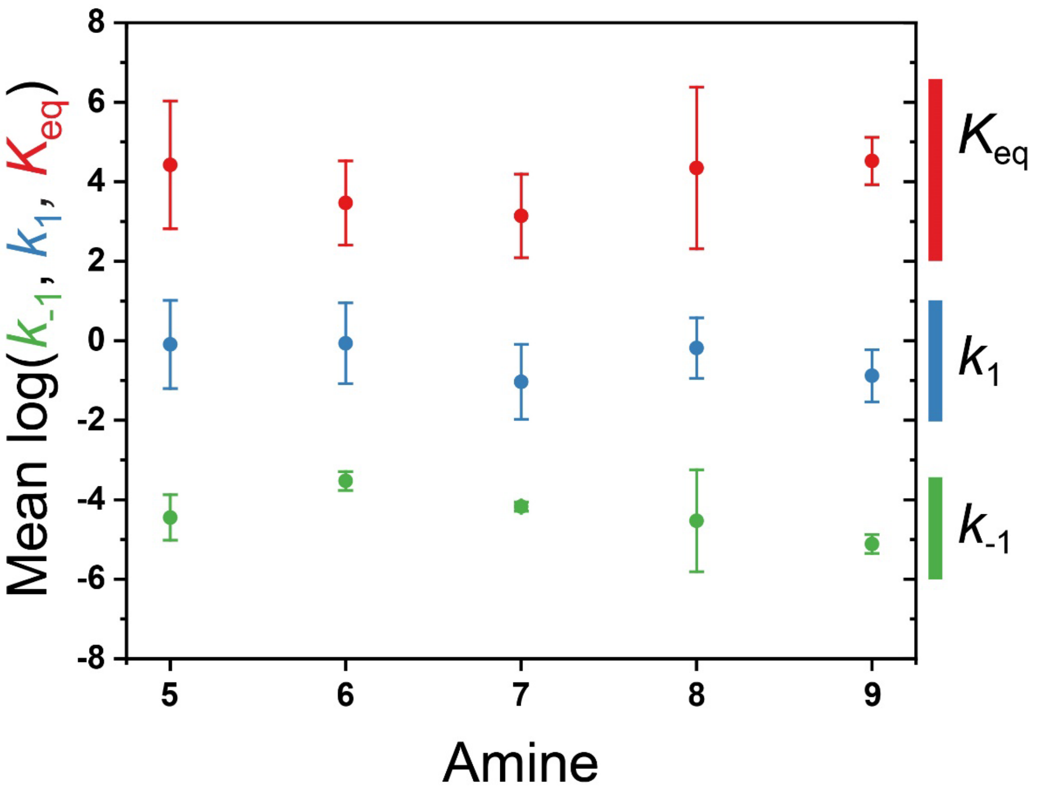

Figure S10. Comparison of the mean rate and equilibrium constants (circles, bars represent the standard deviation) for all aldehyde-amine pairs grouped by amine to enable visual inspection of their relative magnitudes and spread. Statistical analysis was performed using a one-way ANOVA with Tukey's multiple comparisons test, and no statistically significant difference between amines was found at a level of $P < 0.05$.

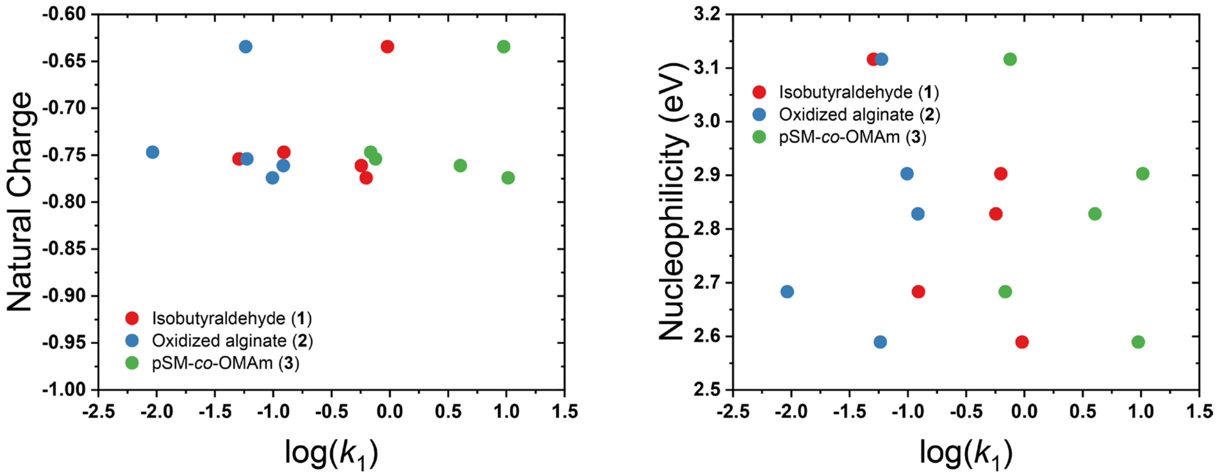

Figure S11. No clear trends were observed between the calculated nucleophilicity or natural charge of our amine series and the corresponding experimentally determined k_1's.

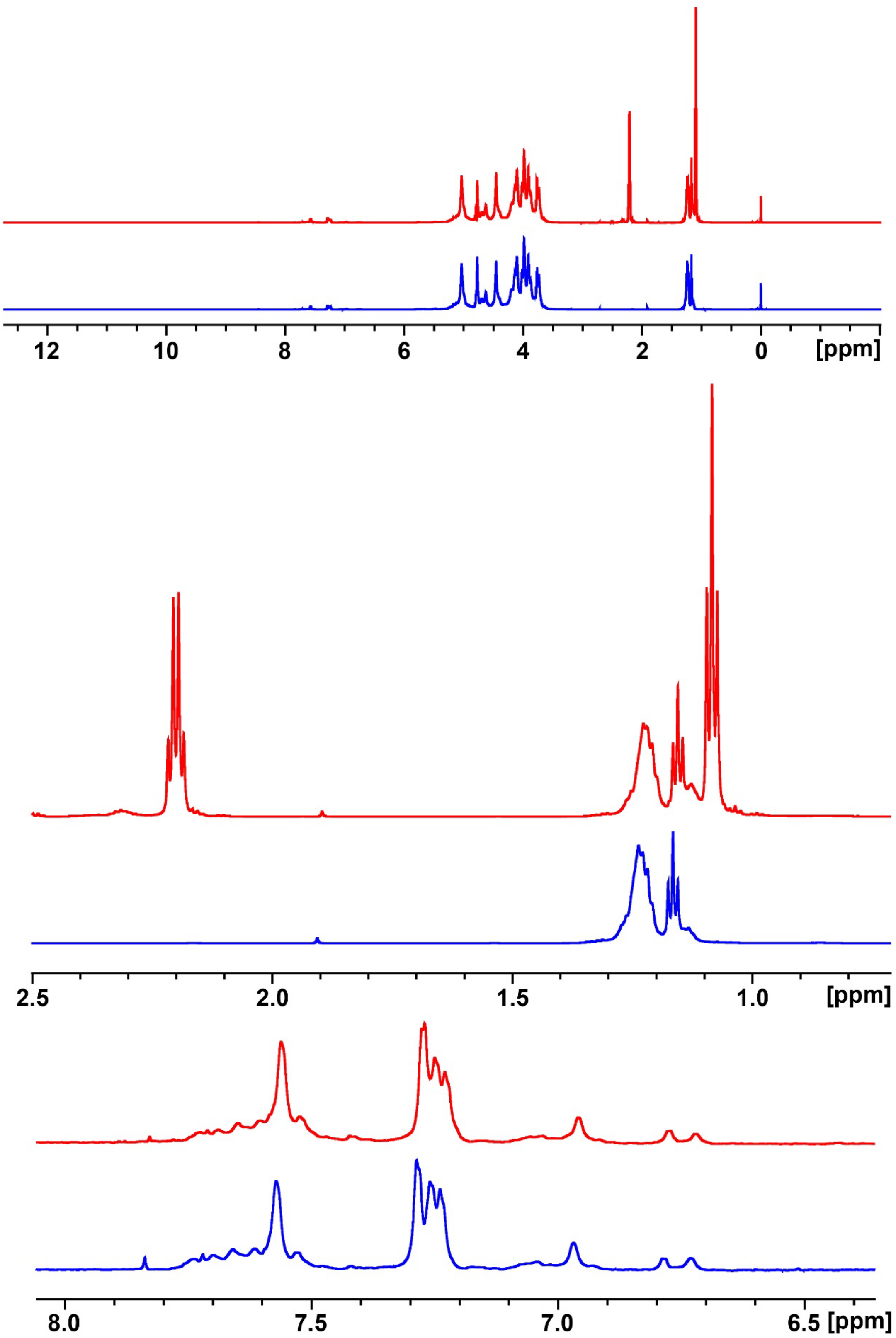

Figure S12. Quantification of the equilibrium imine concentration for the competitive (0.5:0.5, red traces) and non-competitive (0.5:0.0, blue traces) hydrogel systems presented in **Figure 6D**. The integration values and corresponding concentrations are given in **Table S3**. (Top) An overview of the full spectrum, (Middle) a zoom-in in on the methyl (and 7* methylene) regions, and (Bottom) a zoom-in on the imine region. Both spectra are scaled to the internal DSS-d_6 peak and overlap tightly with both the internal standard and alginate backbone.

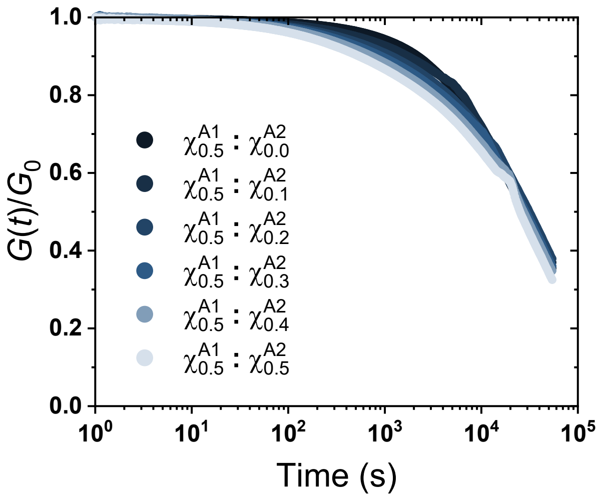

Figure S13. Stress relaxation behavior of mixed hydrogels of **2**+**5***+**7***. We observe that the incremental addition of **7*** increases the short-term stress relaxation while having negligible effect on the long-term stress relaxation.

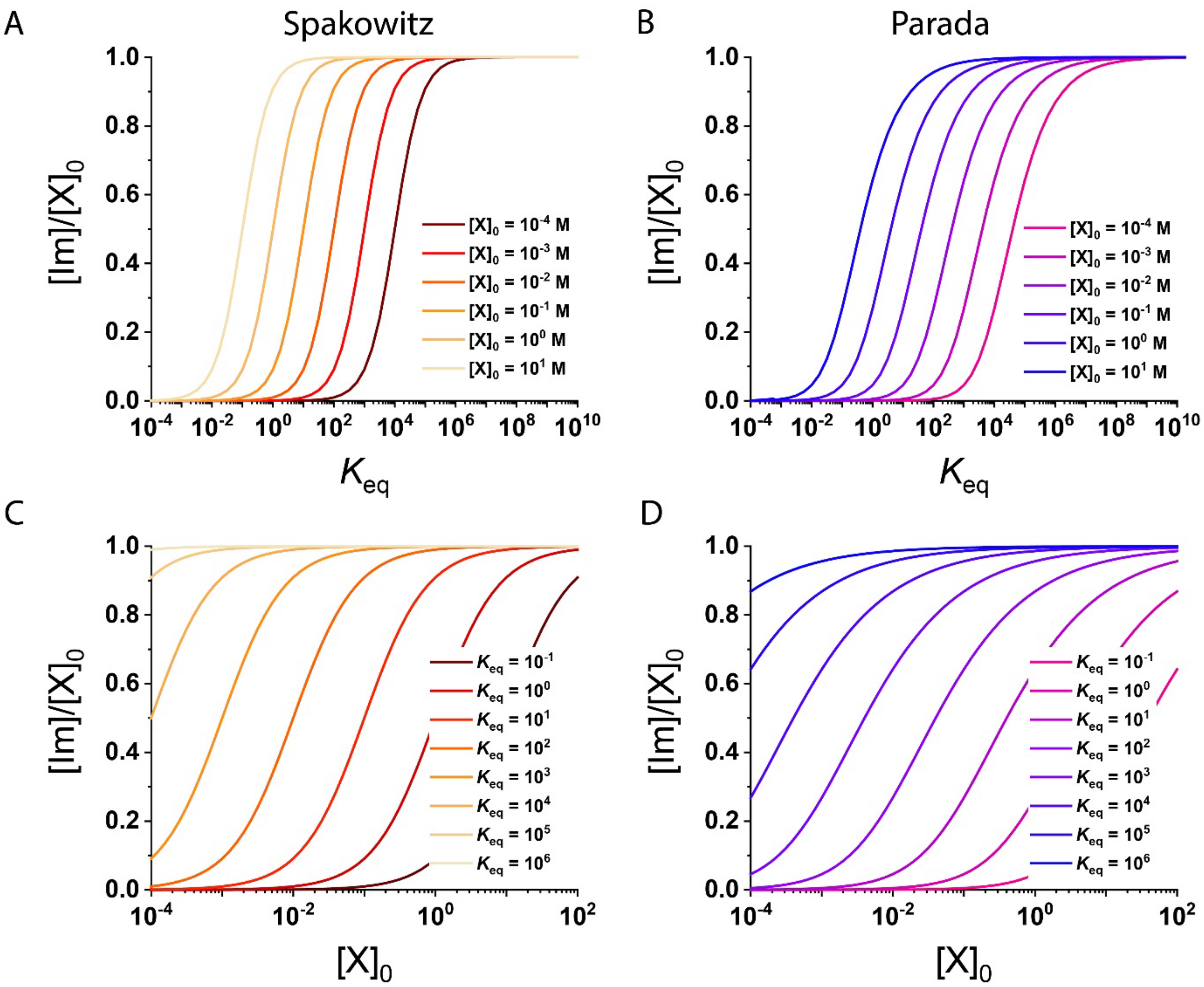

Figure S14. Model plots comparing the shape of the binding isotherm (A–B) and bound fraction *versus* concentration (C–D) produced using the Poisson approach from Spakowitz and colleagues (A & C) or the Bell model approach from Parada and colleagues (B & D).

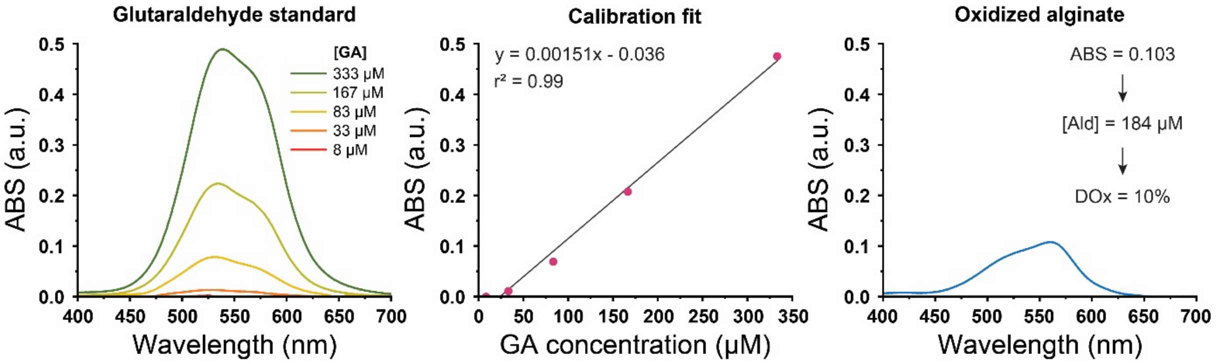

Figure S15. Quantification of the degree of oxidation (DOx) of sodium alginate. Note that glutaraldehyde is bifunctional, as is an oxidized alginate monosaccharide unit, so the calculated aldehyde concentration of 184 µM corresponds to 92 µM of oxidized uronic acid units (10%).

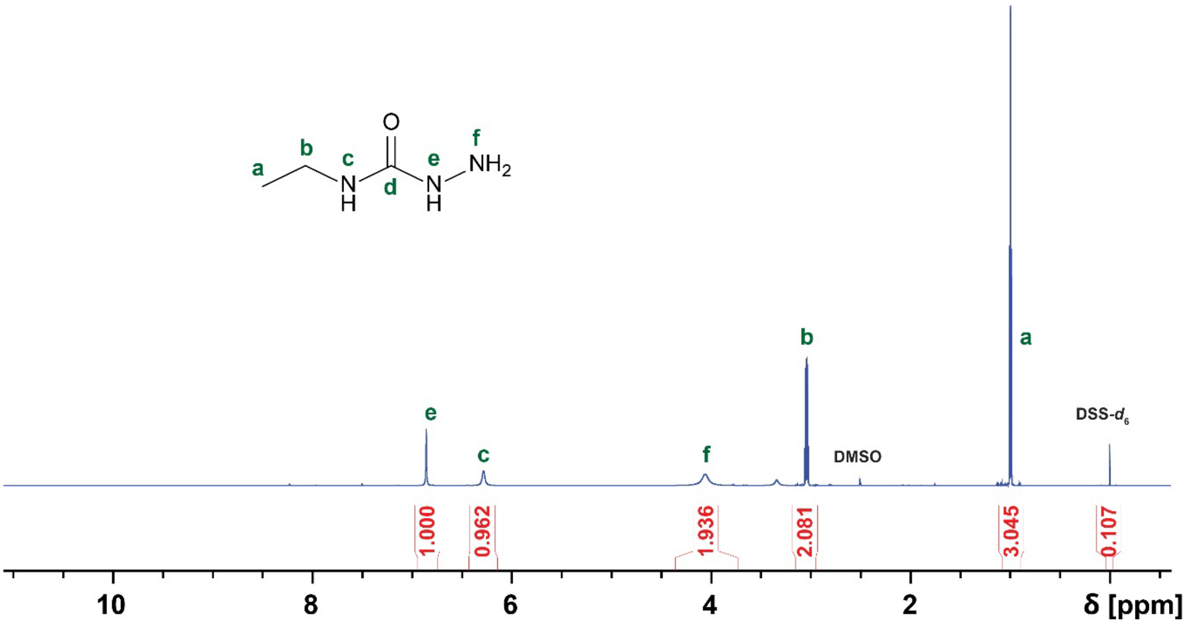

Figure S16. ^{1}H NMR (700 MHz, DMSO-d_6) spectrum of **9**.

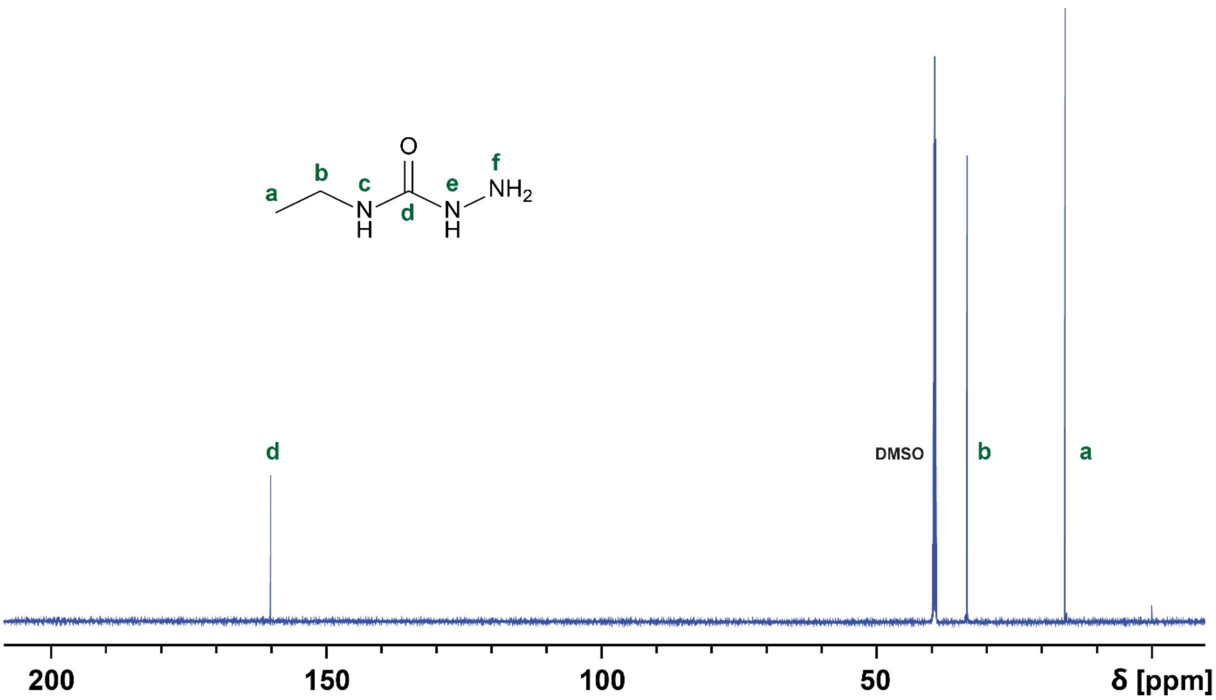

Figure S17. ^{13}C NMR (700 MHz, DMSO-d_6) spectrum of **9**.

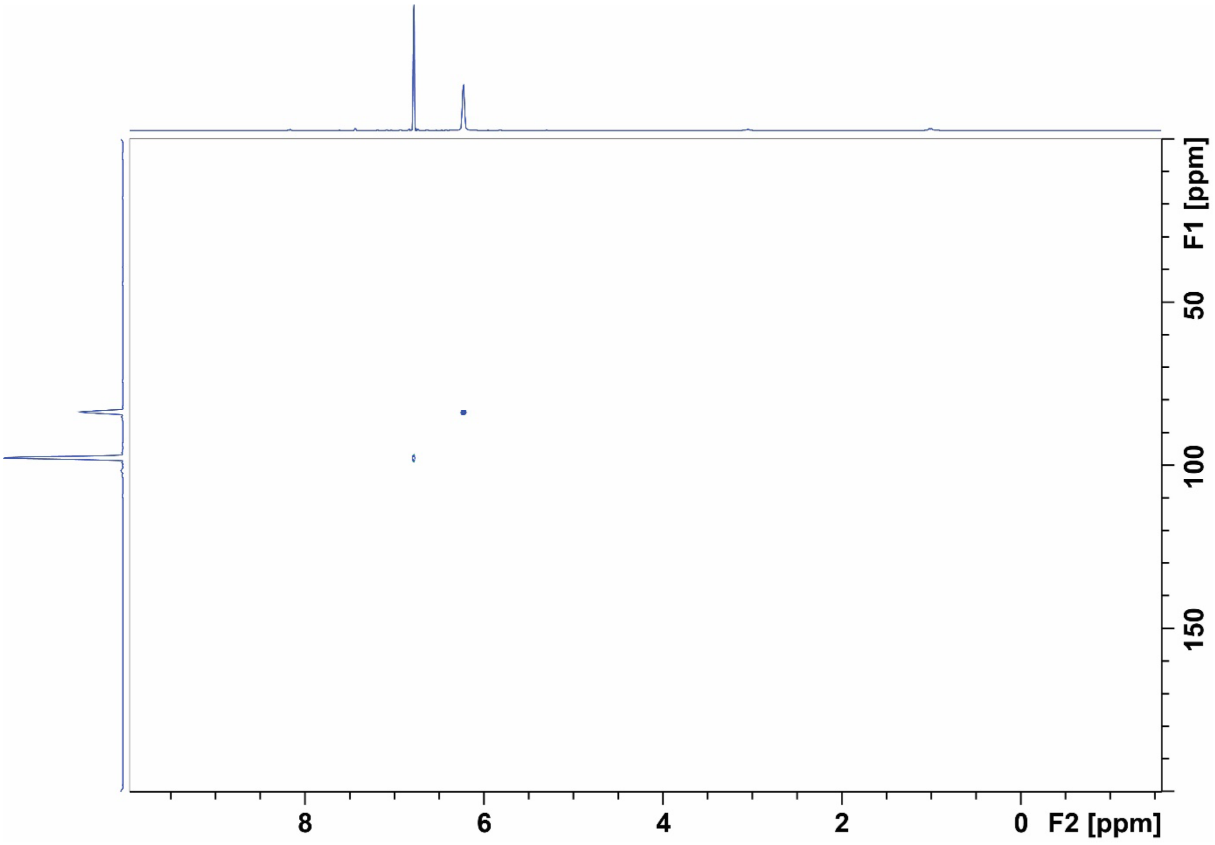

Figure S18. ^1H–^{15}N HSQC NMR (700 MHz, DMSO-d_6) spectrum of **9**.

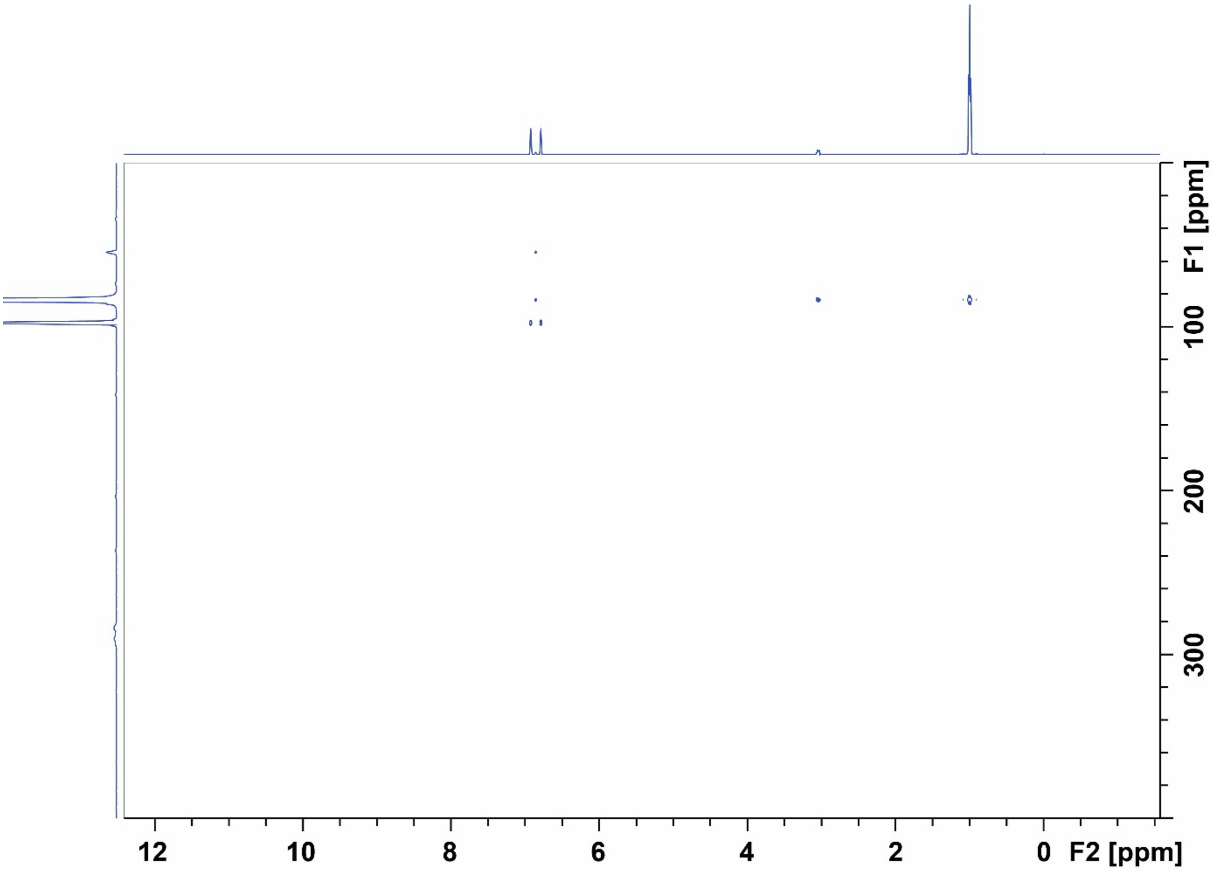

Figure S19. ^1H–^{15}N HMBC NMR (700 MHz, DMSO-d_6) spectrum of **9**.

MODEL FOR MAXIMUM IDEAL CROSSLINKED ALDEHYDE FRACTION AS A FUNCTION OF EQUILIBRIUM CONSTANT AND MOLE FRACTION OF ADDED AMINE.

The model used in **Figure 6A** to describe the maximum ideal fraction of crosslinked aldehyde ($\chi_{\mathrm{XL}}^{\mathrm{Ald}}$) as a function of the added molar ratio of amine (χ_{Am}) is derived as follows: Considering the equilibrium position of a reversible bimolecular reaction between an amine and an aldehyde, we obtain

$$k_1[\mathrm{Am}][\mathrm{Ald}] = k_{-1}[\mathrm{Im}]$$

Conservation of mass requires

$$[\mathrm{Am}] = [\mathrm{Am}]_0 - [\mathrm{Im}]$$
$$[\mathrm{Ald}] = [\mathrm{Ald}]_0 - [\mathrm{Im}]$$

Substituting $K_{\mathrm{eq}} = k_1/k_{-1}$ and expressing the amine ([Am]) and aldehyde ([Ald]) concentrations in terms of their known initial concentrations and imine concentration gives

$$[\mathrm{Im}] = K_{\mathrm{eq}}([\mathrm{Am}]_0 - [\mathrm{Im}])([\mathrm{Ald}]_0 - [\mathrm{Im}])$$

Distribution and rearrangement yields

$$[\mathrm{Im}]^2 - [\mathrm{Im}]\left([\mathrm{Am}]_0 + [\mathrm{Ald}]_0 + \frac{1}{K_{\mathrm{eq}}}\right) + [\mathrm{Am}]_0[\mathrm{Ald}]_0 = 0$$

We now have a quadratic equation with two potential solutions; only one of them has any physical significance

$$[\mathrm{Im}] = \frac{[\mathrm{Am}]_0 + [\mathrm{Ald}]_0 + \frac{1}{K_{\mathrm{eq}}} - \sqrt{\left(-\left([\mathrm{Am}]_0 + [\mathrm{Ald}]_0 + \frac{1}{K_{\mathrm{eq}}}\right)\right)^2 - 4[\mathrm{Am}]_0[\mathrm{Ald}]_0}}{2}$$

We then consider the fraction of imine (with respect to the initial amine concentration) at equilibrium to be equivalent to the probability that an imine is formed from an amine.

$$P_{\mathrm{Im}} = \frac{[\mathrm{Im}]}{[\mathrm{Am}]_0}$$

Assuming ideal crosslinking requires the concomitant formation of two imines, we can then consider the fraction of amines participating in a crosslink to be the square of this probability.

$$\chi_{\mathrm{XL}}^{\mathrm{Am}} = (P_{\mathrm{Im}})^2$$

Finally, multiplication by the molar ratio of initial amines with respect to the initial aldehyde concentration gives us the maximum fraction of aldehydes participating in ideal crosslinks, **Equation 1**.

$$\chi_{\mathrm{XL}}^{\mathrm{Ald}} = \frac{[\mathrm{Am}]_0}{[\mathrm{Ald}]_0}\left(\frac{[\mathrm{Im}]}{[\mathrm{Am}]_0}\right)^2 = \chi_{\mathrm{Am}}\left(\frac{[\mathrm{Im}]}{[\mathrm{Am}]_0}\right)^2$$

Data were generated using an aldehyde concentration of 10.1 mM and an amine concentration ranging from 0–1.4 equiv w.r.t. aldehyde. Data and plots were generated in Origin 2018 (OriginLab®).
